# Distinct clusters of bacterial and fungal microbiota in end-stage liver cirrhosis correlate with antibiotic treatment, intestinal barrier impairment, and systemic inflammation

**DOI:** 10.1080/19490976.2025.2487209

**Published:** 2025-04-21

**Authors:** Laura Buttler, David A. Velázquez-Ramírez, Anja Tiede, Anna M. Conradi, Sabrina Woltemate, Robert Geffers, Birgit Bremer, Vera Spielmann, Julia Kahlhöfer, Anke R.M Kraft, Dirk Schlüter, Heiner Wedemeyer, Markus Cornberg, Christine Falk, Marius Vital, Benjamin Maasoumy

**Affiliations:** a Department of Gastroenterology, Hepatology, Infectious Diseases and Endocrinology, Hannover Medical School, Hannover, Germany; b Institute for Medical Microbiology and Hospital Epidemiology, Hannover Medical School, Hannover, Germany; c German Center for Infection Research (DZIF), Hannover-Braunschweig, Germany; d Genome Analytics Research Group, Helmholtz Centre for Infection Research, Braunschweig, Germany; e Cluster of Excellence RESIST (EXC 2155), Hannover Medical School, Hannover, Germany; f German Center for Infectious Disease Research (DZIF), HepNet Study-House/German Liver Foundation, Hannover, Germany; g Centre for Experimental and Clinical Infection Research, A Joint Venture Between Helmholtz-Centre for Infection Research and Hannover Medical School, TWINCORE, Hannover, Germany; h Center for Individualized Infection Medicine (CiiM), Hannover, Germany; i Institute of Transplant Immunology, Hannover Medical School, Hannover, Germany

**Keywords:** Intestinal microbiome, fungi, dysbiosis, liver cirrhosis, metagenomics, gut barrier, leaky gut, systemic inflammation, cirrhosis-related complications

## Abstract

Decompensated liver cirrhosis (dLC) is associated with intestinal dysbiosis, however, underlying reasons and clinical consequences remain largely unexplored. We investigated bacterial and fungal microbiota, their relation with gut barrier integrity, inflammation, and cirrhosis-specific complications in dLC-patients. Competing-risk analyses were performed to investigate clinical outcomes within 90 days. Samples were prospectively collected from 95 dLC-patients between 2017 and 2022. Quantitative metagenomic analyses clustered patients into three groups (G1–G3) showing distinct microbial patterns. G1 (*n* = 39) displayed lowest diversity and highest *Enterococcus* abundance, G2 (*n* = 24) was dominated by *Bifidobacteria*, G3 (*n* = 29) was most diverse and clustered most closely with healthy controls (HC). Of note, bacterial concentrations were significantly lower in cirrhosis compared with HC, especially for G1 that also showed the lowest capacity to produce short chain fatty acids and secondary bile acids. Consequently, fungal overgrowth, dominated by *Candida* spp. (51.63%), was observed in G1. Moreover, G1-patients most frequently received antibiotics (*n* = 33; 86.8%), had highest plasma-levels of Zonulin (*p* = 0.044) and a proinflammatory cytokine profile along with numerically higher incidences of subsequent infections (*p* = 0.09). In conclusion, distinct bacterial clusters were observed at qualitative and quantitative levels and correlated with fungal abundances. Antibiotic treatment significantly contributed to dysbiosis, which translated into intestinal barrier impairment and systemic inflammation.

## GRAPHICAL ABSTRACT

**Figure:**
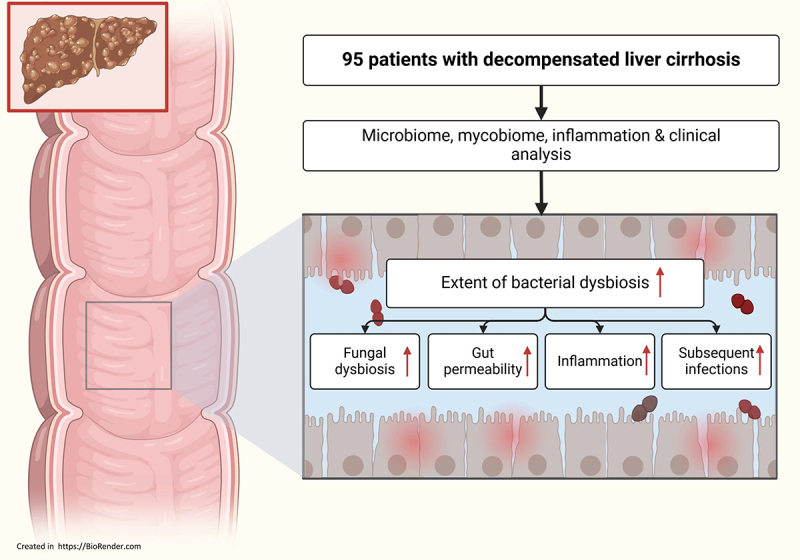

## Introduction

Chronic liver diseases are frequently accompanied by changes of the intestinal microbiome.^1^ Alterations can already be detected in early stages of liver disease and become increasingly aggravated with the progression of hepatic fibrosis.^1,2^ Finally, patients with decompensated liver cirrhosis exhibit disrupted microbiota, characterized by a dramatically increased abundance of potentially pathogenic bacteria and a reduction of commensal microbes.^3^ Particularly, elevated proportions of the pathobionts *Enterococcus*, *Streptococcus*, and *Enterobacteriaceae* have been reported.^4^ Along with a dysbalanced bacterial composition are altered microbiome functions resulting in decreased levels of bacterial key metabolites, such as short chain fatty acids (SCFA) and secondary bile acids (sBA).^5^ SCFA are fermentation end-products with acetate, butyrate, and propionate as the main components that are generated by a myriad of diverse taxa that show decreased abundances in cirrhosis.^6–9^ Physiologically, SCFA feed the epithelium promoting an intact gut barrier and act anti-inflammatory via modulating the immune landscape.^10^ Reduced levels of SCFA-producing bacteria with a concomitant increase of pathobionts impair the intestinal barrier, which may facilitate the translocation of proinflammatory components into the circulation. This may significantly contribute to systemic inflammation in patients with cirrhosis, which is considered to be a key factor for the development of cirrhosis-associated immune dysfunction (CAID), infection susceptibility, hepatic decompensation (e.g. encephalopathy), and acute-on-chronic-liver failure (ACLF).^11–15^ However, causes of intestinal dysbiosis and direct clinical consequences in patients with end-stage liver disease remain largely unexplored as studies including a clinical follow-up remain sparse in this population. Recently, Lehmann and colleagues demonstrated that a lower intestinal diversity with an abundance of either *Enterococcus* or *Enterobacterales* species was linked to a lack of intestinal SCFA and a higher risk for infections in patients with end-stage liver cirrhosis undergoing liver transplantation. Importantly, they also documented that these severe alterations of gut microbiota were not present in all patients with advanced liver cirrhosis.^16^ A relative *Enterococcus* abundance of greater than 20% was observed in 40% of patients, while in about 25% of patients’ microbiota diversity was not different from healthy individuals.^16^

However, several questions remain unaddressed, so far, e.g. reasons causing microbiota dysbiosis among cirrhotic patients still need to be determined. One of the obvious factors that requires attention is concomitant medication.^5^ The frequent use of antibiotics to treat or prevent infections in these patients may, as suggested earlier, induce collateral damage on gut microbiota. Hence, the influence of frequently prescribed drugs on the intestinal microbes needs to be determined in the setting of end-stage liver disease.^5^ Furthermore, bacteria are not the only members of intestinal microbiota. Recently, the role of the mycobiome has gained attention in the context of liver disease and reduced bacterial diversity has also been linked to dysbiotic compositions of intestinal fungi.^17^ However, only little is known about the relevance of fungal dysbiosis in advanced cirrhosis and how it influences the clinical course. Mycotic infections, such as spontaneous fungal peritonitis, are rare, but severe complications in patients with end-stage liver disease and the role of fungi in the context of inflammation is still largely in the dark.^18^

Both treatment of complications and their prevention are essential for patients with decompensated liver cirrhosis. This implicates the question whether distinct microbial features can function as a novel tool in risk stratification and as a therapeutic target in clinical management. Therefore, it is vital not only to identify unfavorable microbial features but to gain a detailed understanding of causes of dysbiosis and pathophysiological consequences. Our study provides novel quantitative insights into gut microbiota in the setting of end-stage liver disease. We investigated the impact of medication on the interplay between bacterial as well as fungal components of gut microbiota and how this affects the clinical phenotype of patients with decompensated liver cirrhosis, particularly focusing on inflammation and cirrhosis-associated complications.

## Results

### Clinical characteristics of the overall study cohort

Median age was 57.5 years and 73.7% were male patients (*n* = 70). Median baseline Model for End-Stage Liver Disease (MELD) was 16 (Table 1). Most common cause of cirrhosis was alcohol-related (*n* = 61; 64.2%). At the time point of stool collection, 8.4% of the patients (*n* = 8) was diagnosed with spontaneous bacterial peritonitis (SBP), almost a third (*n* = 28; 29.5%) had any infection, 14.7% had acute-on-chronic liver failure (ACLF; *n* = 14) and 15.8% (*n* = 15) showed symptoms of overt hepatic encephalopathy (oHE).

**Table 1.** Baseline characteristics. ACLF: Acute-on-chronic liver failure, AIH: Autoimmune hepatitis, ALD: Alcohol-related liver disease, ALT: Alanine aminotransferase, AST: Aspartate aminotransferase, BL: Baseline, CRP: C-reactive protein, gamma-gt: Gamma-glutamyltransferase, INR: International normalized ratio, MASLD: Metabolic dysfunction associated liver disease, MetALD: Metabolic dysfunction- and alcohol-related liver disease, MELD: Model for end-stage liver disease, oHE: Overt hepatic encephalopathy, PBC: Primary biliary cholangitis, PSC: Primary sclerosing cholangitis, SBP: Spontaneous bacterial peritonitis.

	All patients (n = 95)	Group 1 (n = 39)	Group 2 (n = 24)	Group 3 (n = 29)	*p* value*
Age (y)	57.50 (50.89–65.08)	59.41 (51.07–65.33)	56.64 (52.98–60.88)	55.83 (50.63–71.73)	0.715
Sex					0.885
− Male − Female	70 (73.7) 25 (26.3)	29 (74.4) 10 (25.6)	18 (75.0) 6 (25.0)	23 (79.3) 6 (20.7)	
Etiology**					
− ALD − MetALD − MASLD − Viral − PSC − PBC − AIH − cryptogenic − others	47 (49.5) 14 (14.7) 9 (9.5) 6 (6.3) 4 (4.2) 3 (3.2) 7 (7.4) 10 (10.5) 9 (9.5)	18 (46.2) 4 (10.3) 2 (5.1) 3 (7.7) 1 (2.6) 2 (5.1) 2 (5.1) 6 (15.4) 4 (10.3)	14 (58.3) 4 (16.7) 3 (12.5) 1 (4.2) 2 (8.3) 0 (0.0) 2 (8.3) 1 (4.2) 0 (0.0)	13 (44.8) 6 (20.7) 4 (13.8) 1 (3.4) 1 (3.4) 1 (3.4) 3 (10.3) 3 (10.3) 4 (13.8)	0.559 0.483 0.430 0.710 0.530 0.537 0.716 0.379 0.187
MELD	16 (12–22)	18 (14–24)	15 (12–20)	12 (10–14)	0.004
SBP at BL	8 (8.4)	3 (7.7)	1 (4.2)	4 (13.8)	0.445
Infection at BL	28 (29.5)	12 (30.8)	9 (37.5)	7 (24.1)	0.574
ACLF at BL	14 (14.7)	8 (20.5)	4 (16.7)	1 (3.4)	0.125
oHE at BL	15 (15.8)	7 (17.9)	4 (16.7)	4 (13.8)	0.899
Laboratory values					
White blood cells (Tsd/µl)	6.00 (3.80–8.80)	6.30 (3.65–10.55)	5.70 (3.08–8.18)	4.60 (4.20–8.10)	0.703
Hemoglobin (g/dl)	9.40 (8.30–11.20)	9.00 (7.80–9.80)	9.35 (8.65–10.32)	10.30 (8.50–11.60)	0.052
Platelets (Tsd/µl)	103.00 (61.00–173.00)	94.00 (57.50–145.00)	85.50 (61.25–201.25)	117.00 (88.00–152.00)	0.310
INR	1.31 (1.16–1.54)	1.48 (1.27–1.62)	1.38 (1.20–1.51)	1.16 (1.10–1.32)	0.001
Potassium (mmol/l)	4.10 (3.80–4.60)	4.20 (3.85–4.60)	3.90 (3.70–4.32)	4.30 (3.90–4.60)	0.259
Sodium (mmol/l)	135.00 (138.00)	134.00 (131.00–136.00)	136.00 (132.75–139.00)	134.00 (130.00–137.00)	0.294
Bilirubin (µmol/l)	26.00 (14.00–93.00)	55.00 (18.50–134.50)	21.00 (15.00–46.75)	17.50 (11.75–44.75)	0.021
Creatinine (µmol/l)	115.00 (89.75–143.00)	115.00 (96.50–139.00)	119.00 (88.75–144.75)	114.50 (80.50–139.50)	0.815
AST (U/l)	41.00 (30.00–66.00)	46.00 (33.00–75.50)	39.50 (33.00–53.00)	41.00 (32.00–82.00)	0.683
ALT (U/l)	24.50 (18.00–42.75)	26.50 (19.25–44.25)	25.50 (17.75–32.50)	24.00 (17.00–52.00)	0.960
AP (U/l)	138.00 (99.00–197.00)	134.00 (98.00–168.00)	152.00 (92.75–220.25)	162.00 (116.00–220.00)	0.182
Gamma-GT (U/l)	112.00 (58.00–212.00)	90.00 (43.50–170.50)	151.00 (73.00–257.50)	142.00 (71.00–259.00)	0.041
CRP (mg/l)	16.80 (7.90–29.10)	21.40 (9.40–32.40)	11.50 (6.05–24.75)	14.20 (8.15–21.65)	0.183
Serum-cholinesterase (kU/l)	2.08 (1.30–3.07)	1.54 (1.18–2.20)	2.09 (1.50–2.96)	2.21 (1.37–3.26)	0.183
Albumin (g/l)	30.00 (25.50–35.50)	28.00 (26.00–31.00)	33.00 (25.00–36.00)	28.00 (27.00–33.00)	0.467
Diabetes mellitus	25 (26.3)	7 (17.9)	8 (33.3)	9 (31.0)	0.307
Active alcohol consumption	9 (9.9)	2 (5.6)	2 (8.3)	5 (17.9)	0.256
Medication					
Any antibiotics within one week before BL	68 (71.6) (73.9)	33 (86.8)	19 (79.2)	15 (51.7)	0.004
Norfloxacin within 1 week before BL	11 (11.6) (73.3)	7 (18.9)	1 (4.2)	3 (10.3)	0.213
Rifaximin within 1 week before BL	39 (41.1) (92.9)	14 (35.9)	16 (66.7)	8 (27.6)	0.011
Any other antibiotics − Vancomycin − Meropenem − Ampicillin/Sulbactam −Piperacillin/Tazobactam − Ceftriaxone − Clindamycin	39 (41.1) 6 (6.3) 7 (7.4) 7 (7.4) 10 (10.5) 11 (11.6) 3 (3.2)	28 (71.8) 6 (15.4) 5 (12.8) 5 (12.8) 8 (20.5) 9 (23.1) 1 (2.6)	4 (16.7) 0 (0.0) 1 (4.2) 1 (4.2) 1 (4.2) 1 (4.2) 0 (0.0)	7 (24.1) 0 (0.0) 1 (3.4) 1 (3.4) 1 (3.4) 1 (3.4) 2 (6.9)	<0.001 0.013 0.269 0.269 0.039 0.019 0.353
Number of different antibiotics	1.00 (0.00–2.00)	2.00 (1.00–2.50)	1.00 (0.00–1.00)	1.00 (0.00–1.00)	<0.001
Non selective beta blockers at BL	50 (52.6)	19 (50.0)	13 (54.2)	16 (55.2)	0.904
Carvedilol	41 (43.6)	18 (47.4)	11 (45.8)	11 (37.9)	0.726
Propranolol	9 (9.6)	1 (2.6)	2 (8.3)	5 (17.2)	0.112
Proton pump inhibitors at BL	76 (80.9)	32 (84.2)	18 (75.0)	24 (82.8)	0.644
Lactulose at BL	59 (62.8)	23 (60.5)	19 (79.2)	15 (51.7)	0.114
Ornithine aspartate at BL	28 (29.8)	8 (21.1)	12 (50.0)	8 (27.6)	0.050

Values as number (percentage) or median (interquartile range).

*Chi-Square was used for comparison of categorical variables, Fisher–Freeman–Halton´s exact for categorial covariables with less than five cases/field. Kruskal–Wallis test was performed for continuous values.

**Results in more than 100% due to mixed etiology.

### Gut bacteria clustered into three compositionally distinct groups that varied in concentrations and in abundances of key functions

**Figure 1.**
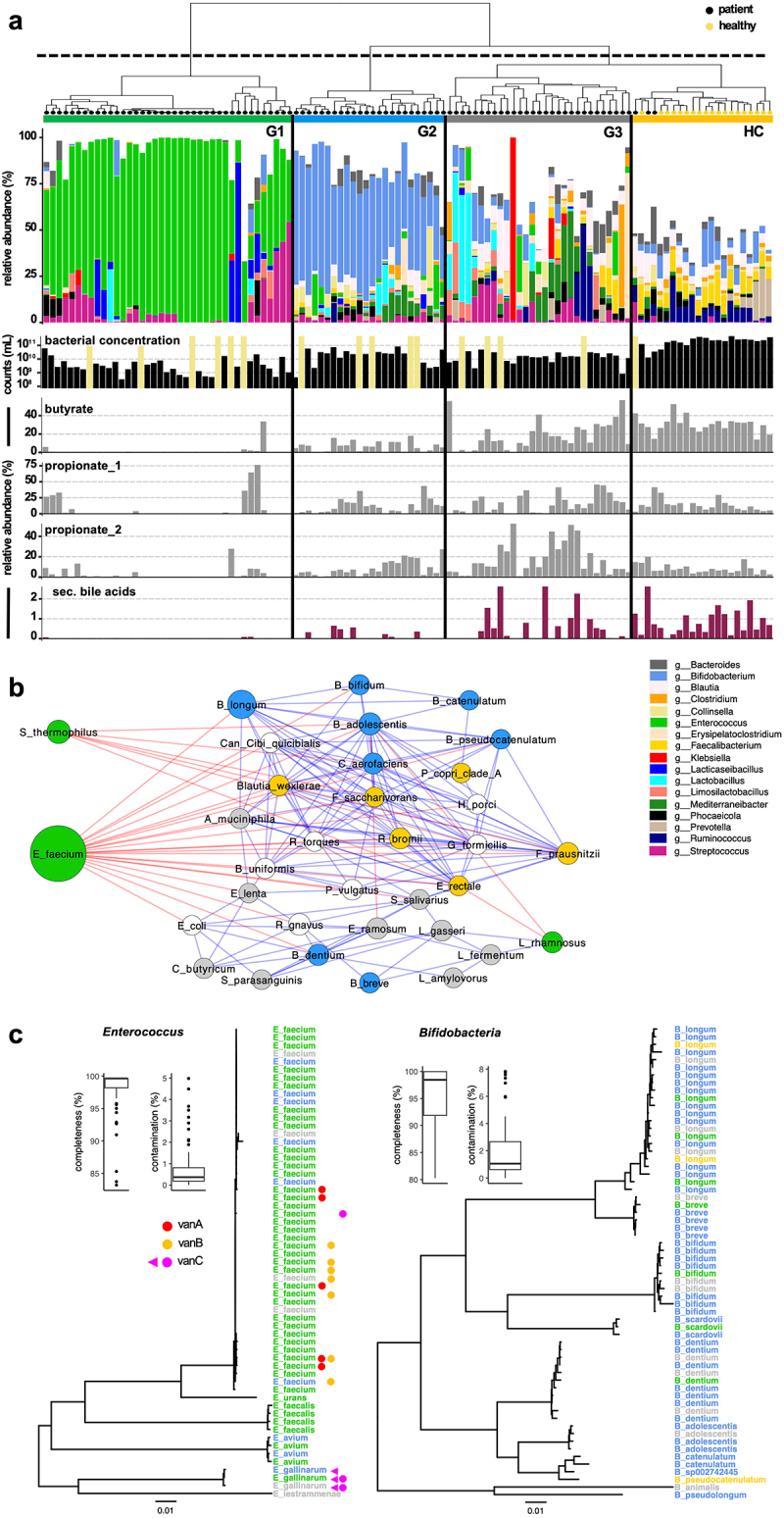
Bacterial communities of patients and healthy controls (HC). Panel (a) Shows hierarchical clustering result along with composition on the genus level for all samples yielding three distinct patient groups (G1–G3) and a separate HC group. Below, bacterial cell concentrations measured by flow cytometry (bacterial concentration; khaki color indicates no data available) as well as relative abundances of pathways for the synthesis of butyrate, propionate (two pathways) and secondary bile acids are given. In panel (b) Network of bacterial species based on correlation analyses is given, where node size refers to mean abundance of each species and colors of edges represent positive (blue) and negative (red) correlations. Species most abundant in a specific group are color coded. Panel (c) Shows phylogenetic trees for genomes of *Enterococcus* and *Bifidobacteria* assembled from metagenomes of patient samples along with their basic quality parameters completeness and contamination. Group origins of genomes are indicated by color and presence of genes encoding vancomycin resistance (*vanABC)* in respective samples is given; The violet triangle signifies *vanC* detection on genomes.

Diversity and composition of gut bacteria varied widely between patients and hierarchical clustering suggested three groups that were separated from healthy controls (HC). Group one (G1; *n* = 39) was dominated by *Enterococcus* sp. (median abundance of 77.97% (51.67–95.44)), whereas *Bifidobacteria* were most abundant in group two (G2; *n* = 24) (52.31% (42.82–65.52)) (Figure 1a; Supplementary figure S1). The third group (G3; *n* = 29) clustered most closely with HC and showed a diverse pattern; three patient samples were located within the HC-group. Ordination analyses verified the observed clusters (Supplementary figure S2). Furthermore, PERMANOVA analyses demonstrated significant differences (*p* < 0.01) between patient groups as well as between patients and HC. Diversity based on observed species and the Shannon index was greatly distinct between groups, where G1 showed lowest values of 10 (8–17) (observed species) and 0.74 (0.22–1.37) (Shannon) followed by G2 (56 (42–79); 2.17 (1.98–2.62)) and G3 (62 (44–87); 2.72 (2.23–3.02)); results based on the Simpson and Inverted Simpson index supported those findings (Supplementary figure S1d-e). HC displayed the highest diversities with 261 (200–313) species and a Shannon index of 3.94 (3.68–4.28) (Figure 1a; Supplementary figure S1d-e). Bacterial concentrations determined by flow cytometry followed this pattern: G1 was characterized by a low bacterial load of 2.65 × 10^9^ cells/gram stool (1.21 × 10^9^–6.67 × 10^9^), which was 100 times lower compared with HC (2.38 × 10^11^ cells/gram stool (1.71 × 10^11^–3.16 × 10^11^). G2 and G3 showed higher values of 2.55 × 10^10^ cells/gram stool (5.52 × 10^9^–3.77 × 10^10^) and 1.52 × 10^10^ cells/gram stool (7.40 × 10^9^–2.65 × 10^10^), respectively. However, bacterial concentrations in G2 and G3 were still markedly below those measured in HC. Similar results were observed for abundances of microbial key pathways encoding enzymes for the synthesis of the SCFA butyrate and propionate as well as for secondary bile acids (sBA) (Figure 1a). Genes associated with butyrate and sBA were almost absent in G1 and also lower in G2 (6.47% (1.06–10.59) and 0.00% (0.00–0.20)) and G3 (13.02% (2.27–24.29) and 0.03% (0.00–0.71)) compared with HC, where 31.49% (26.57–33.49) and 0.54% (0.33–1.21), respectively, of calculated genomes harbored genes of those pathways (Figure 1a; Supplementary figure S3). Propionate pathways had higher values in patient groups G2 (pdiol: 5.82% (2.47–14.26), suc: 8.66% (2.94–16.48)), and G3 (pdiol: 10.49% (5.30–26.31), suc: 13.02% (2.27–24.29)) displaying similar concentrations as those observed in HC (pdiol: 7.04 (5.03–8.69), suc: 12.12 (6.53–16.57)); with a few exceptions those pathways were lower in samples derived from G1.

Correlation analyses revealed separate clustering of signature taxa of individual groups. *E. faecium*, which was the main *Enterococcus* species, was negatively associated with a variety of other taxa such as G2’s *Bifidobacteria* species that, apart from *B. breve* and *B. dentium*, clustered closely together (Figure 1b). Other taxa specific for G1, namely, *Streptococcus thermophilus* also correlated negatively with several taxa of G2 and HC. *Faecalibacterium prausnitzii*, which was detected at highest abundances in HC, formed a counterpart that was positively associated with a variety of other taxa prevalent in HC as well as with some members most abundant in G2 and G3, whose taxa formed overall separated groups (Figure 1b).

In total, we assembled 645 high-quality genomes of patient samples, where 59 and 62 genomes were annotated as *Enterococcus* and *Bifidobacteria*, respectively (Figure 1c). The former was primarily derived from samples of G1, where genomes were retrieved from 37 (94.9%) samples and the bulk was annotated as *E. faecium*. In five samples, two genomes of this taxon were obtained (one sample harbored three *Enterococci* genomes) with an average completeness of 97.79% ± 3.84% and a contamination of 0.84% ± 1.17%. Genes associated with Vancomycin resistance were detected in 14 samples (11 (38%) in G1). However, apart from vanC in all three *E. gallinarum* strains, *E. faecium* genomes were devoid of *vanA, B* genes, most probably due to their presence on plasmids. For *Bifidobacteria*, genomes from several distinct species were assembled that primarily derived from samples of G2. Their average completeness was 95.59% ± 5.55% with a contamination of 2.07% ± 2.21% and up to four distinct genomes were detected in one sample.

### Abundances of fungi were greatly distinct between patient groups and correlated with specific bacterial taxa

**Figure 2.**
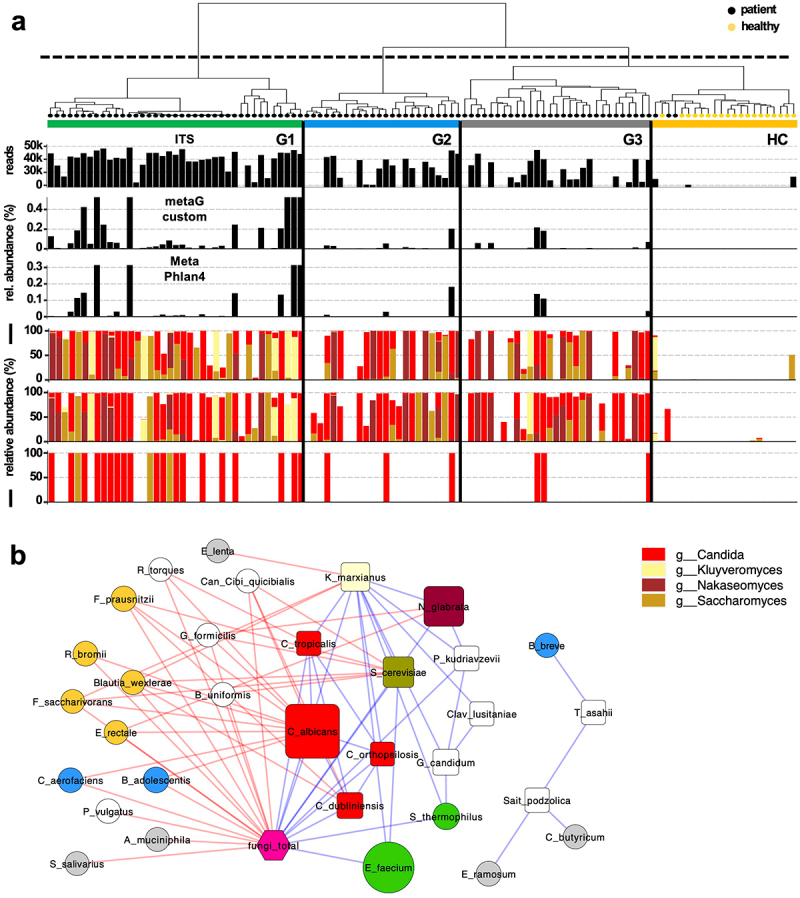
Fungal communities of patients and healthy controls (HC). Results in panel (a) are displayed according to groupings based on hierarchical clustering result of bacterial communities that are shown in Figure 1. Three different methods were applied based on (i) Amplification of ITS region, (ii) Custom metagenomic analysis and (iii) MetaPhlan4. Fungal concentrations measured by reads associated with fungi derived from the individual methods are given as black bars, whereas composition on the genus level is given in the three barplots below (same order as above). In panel (b) A network of fungal species and associated bacterial species based on correlation analyses is given, where node size refers to mean abundance of each species and colors of edges represent positive (blue) and negative (red) correlations. Bacterial species primarily associated with a specific group are color coded (compare Figure 1).

We analyzed fungal organisms based on three methods, namely, (i) amplification and sequencing of the internal transcribed spacer (ITS) region and two metagenome-based analyses comprising (ii) a custom workflow based on mapping reads to a comprehensive fungal database involving a multitude of fungal genes, and (iii) MetaPhlan4 that uses specific single copy marker genes. Results between ITS and the custom metagenomic approach were largely congruent and detected much higher fungal abundances in G1 compared with other groups (Figure 2a; Supplementary figure S1). Samples of HC were largely devoid of fungal communities. Four genera dominated fungal compositions, with species belonging to *Candida* spp. being the most abundant ((median relative abundance of 51.63% (6.53–95.45)). Fungi of the related genus *Nakaseomyces* (0.00% (0.00–13.24)) and of the family *Saccharomycetaceae, Saccharomyces* (0.12% (0.0–7.00)), and *Kluyveromyces* (0.00% (0.00–0.01)) were detected in patients as well, where results of ITS and the custom bioinformatic approach yielded congruent insights (Figure 2a). Results based on MetaPlan4 supported the general trend of G1 being the group most colonized by fungi; however, the method could only detect taxa of the genera *Candida* and *Saccharomyces*, and showed lower sensitivities.

Correlation analyses between bacterial and fungal species revealed a clear pattern, with total concentrations of fungi being positively correlated only with bacterial taxa of G1 (*E. faecium* and *S. thermophilus*), whereas bacteria of samples from other patient groups were all negatively correlated with total fungi and individual fungal species. A few species (*n* = 5) formed a separate module (Figure 2b).

To verify the observed fungal patterns, we reanalyzed metagenomic data from a previous study investigating microbiota in cirrhotic individuals.^5^ Based on bacterial composition on the genus-level samples clustered comparable to our cohort, however, the groups dominated by *Enterococcus* and *Bifidobacteria* comprised less patients, namely, *n* = 35 (15%) and *n* = 37 (16%) of all patients, respectively (Supplementary figure S4). The same four fungal genera were dominating, and fungal concentrations were the highest in the *Enterococcus* enriched group. Similar to our data *Candida* spp. was the most abundant fungal taxon in this group (53.38% (7.03–98.07)) (Supplementary figure S4). A few other samples not linked to *Enterococcus* displayed high levels of *Candida* spp. as well. Furthermore, the study additionally measured certain key metabolites and we correlated their concentrations with calculated abundances of respective pathways based on metagenomes. All pathways correlated with metabolites, especially in the case of butyrate (0.72 (Pearson) and 0.67 (Spearman)) and sBA (0.44 (Pearson) and 0.73 (spearman)), demonstrating that metagenomic-based analyses enable inferring concentrations for those compounds.

### Antibiotic treatment significantly contributed to distinct intestinal microbiota compositions

No differences in age and cirrhosis etiology were detected between groups, whereas median MELD was higher in G1 than in the other groups. Administration of frequently prescribed drugs, e.g. nonselective beta blockers and proton pump inhibitors, was almost equally distributed over the patient cohort (Table 1). Furthermore, dietary and lifestyle habits were comparable between groups (Table 2).

**Table 2.** Diet and lifestyle of the study cohort.

	All patients (n = 86)	Group 1 (n = 35)	Group 2 (n = 24)	Group 3 (n = 27)	*p* value*
Coffee drinkers	57 (66.3)	25 (71.4)	13 (54.2)	19 (70.4)	0.334
Smokers	31 (32.0)	12 (32.4)	11 (45.8)	8 (29.6)	0.432
Vegetarian	5 (5.8)	2 (6.1)	1 (4.2)	2 (7.4)	0.887
>2 fruits/day	22 (26.5)	7 (21.9)	7 (29.2)	8 (29.6)	0.750
vegetable intake: ≥ 5 days/week	45 (60.8)	20 (66.7)	10 (50.0)	15 (62.5)	0.486

Values as number (percentage). *Chi-Square test.

A high proportion of 71.6% (*n* = 68) received antibiotic treatment within 1 week before stool was provided (Table 1). The number of patients with intake of any antibiotics differed significantly between groups (*p* = 0.004), with G1 containing the highest quantity of patients with antibiotic treatment (*n* = 33; 86.8%), followed by G2 (*n* = 19; 79.2%). Similarly, the median number of different antibiotics per patient was significantly higher in G1 (*p* < 0.001). In addition, the type of administered antibiotics differed. Besides a significantly higher intake of Ceftriaxone in patients from G1 (*p* = 0.019), the administration of Vancomycin (*p* = 0.013) and Piperacillin/Tazobactam (*p* = 0.039) was also higher in this group, whereas antibiotic treatment in G2 was mainly dominated by Rifaximin (*n* = 16; 66.7%; *p* = 0.011) (Table 1).

In order to get more detailed insights into how antibiotic treatment affected community composition stratified analyses were performed. Comparing untreated samples with HC revealed higher abundances of several taxa including *Enterococcus* and *Streptococcus* spp among others in patient samples, whereas many typical commensals, such as *Faecalibacterium* spp, G*emmiger formicilis*, and *Roseburia faecis*, were higher in HC indicating that cirrhosis *per se* is contributing to dysbiosis (Supplementary figure S5). Patient samples treated with (broad spectrum) antibiotics greatly further enriched for *Enterococcus*, specifically *E. faecium*, whereas *Bifidobacteria* and a few commensals, such as *Blautia* and *Mediterraneibacter*, were decreased upon treatment (Supplementary figure S5). Overall, Bray Curtis dissimilarities to HC of treated samples were on average significantly higher compared with untreated samples (any antibiotics: 0.958 ± 0.061 vs 0.901 ± 0.086; broad spectrum antibiotics: 0.978 ± 0.061 vs 0.931 ± 0.070) demonstrating that antibiotic treatment enhanced dysbiosis (data not shown).

### Abundance of Enterococcus is linked to an increase in surrogate markers of an impaired intestinal barrier and pro-inflammatory cytokines

**Figure 3.**
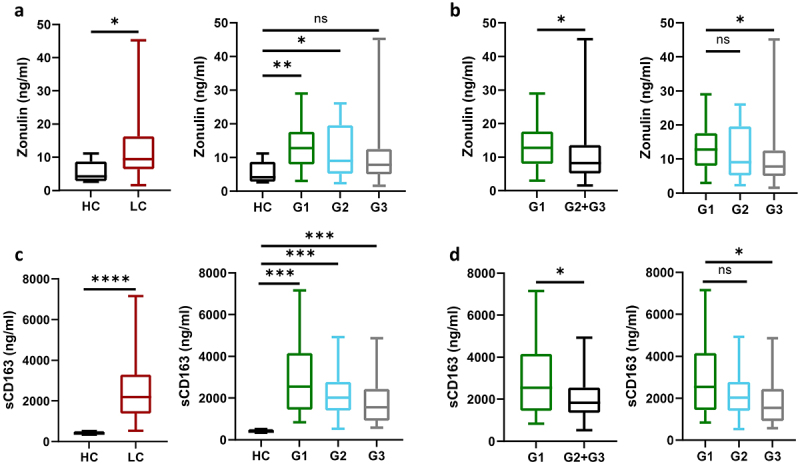
Zonulin levels were higher in patients than in HC (*p* = 0.023) (a) Among cirrhosis patients, G1 displayed significantly higher zonulin concentrations than the other patients (*p* = 0.044) (b) Concentrations of sCD163 in cirrhosis patients exceeded those of HC (*p* < 0.001) (c), with highest values in G1 compared to the other patients (*p* = 0.019) (d).

Significantly greater concentrations of Zonulin were discovered in cirrhosis patients compared to HC (9.5 ng/ml (6.4–16.3) vs. 4.2 ng/ml (2.8–8.7); *p* = 0.023). Patients of G1 reached the highest median Zonulin values that exceeded the measured levels of the other patients significantly (12.8 ng/ml (8.0–17.6) vs. 8.2 ng/ml (5.2–13.6); *p* = 0.044) (Figure 3a,b). Similarly, sCD163 concentrations were significantly elevated in dLC patients contrasted to healthy subjects (2193.0 ng/ml (1377.0–3290.8) vs. 388.0 ng/ml (359.5–507.5); *p* < 0.001). Likewise, G1 exhibited significantly higher sCD163 levels than the other patients (2544.9 ng/ml (1448.4–4151.4) vs. 1836.0 ng/ml (1356.6–2565.3); *p* = 0.019) (Figure 3c,d).

**Figure 4.**
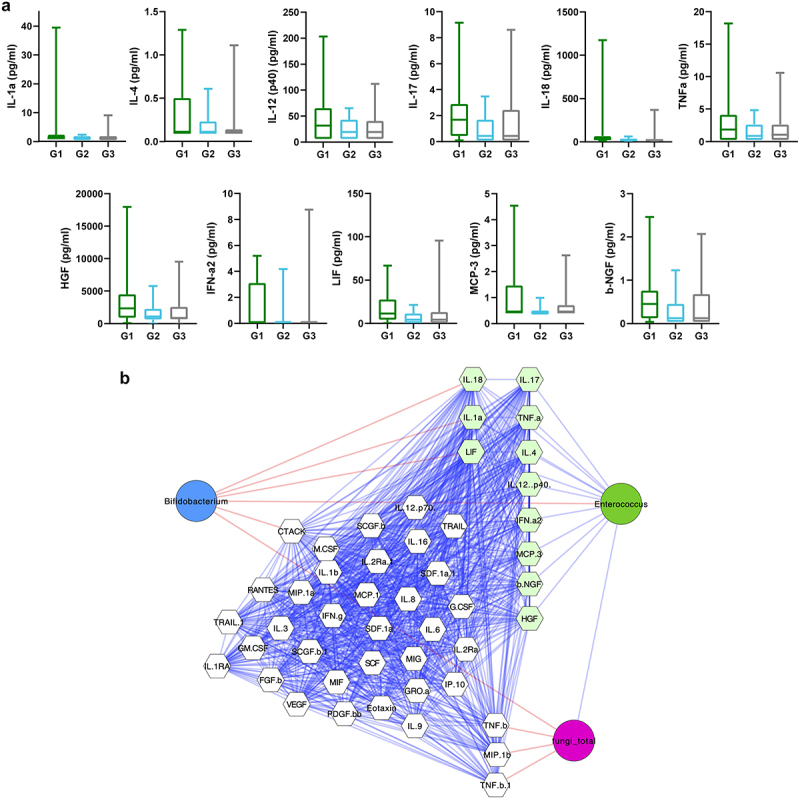
Concentrations of cytokines that differed between groups based on hierarchical clustering on microbiota composition (a). Correlation analyses between cytokines (*n* = 48) and main microbiota components displayed significant correlations between eleven cytokines (light-green) and *Enterococcus* abundance (b). Colors of edges represent positive (blue) and negative (red) correlations.

Procrustes analyses revealed that overall bacterial composition and cytokine patterns significantly correlated (*p* < 0.05). Following pair-wise correlation analyses, we identified 11 cytokines, namely Interleukin (IL)-1alpha, IL-4, IL-12 (p40), IL-17, IL-18, tumor-necrosis factor (TNF)-alpha, hepatocyte growth factor (HGF), Interferon (IFN)-alpha2, leukemia-inhibiting factor (LIF), monocyte-chemotactic protein-3 (MCP-3), and beta-nerve growth factor (b-NGF) that significantly correlated with *Enterococcus* abundance. Median concentrations of these cytokines are depicted in Figure 4. Cytokines showing a correlation with *Enterococcus* were then compared between G1 and the other patients. G1-patients exhibited significantly higher plasma levels of IL-1alpha (*p* = 0.002), IL-4 (*p* = 0.038), IL-17 (*p* = 0.038), IL-18 (*p* = 0.007), HGF (*p* = 0.031), IFN-alpha2 (*p* = 0.015), LIF (*p* = 0.026), MCP-3 (*p* = 0.033), and b-NGF (*p* = 0.037) (Table 3). Concentrations of IL-12 p40 (*p* = 0.075) and TNF-alpha (*p* = 0.057) did only trend to exceed those of other patients (Table 3). Supplementary table S1 shows an overview of measured cytokine concentrations of the entire 48 measured cytokines.

**Table 3.** Plasma concentrations of cytokines that correlated significantly with *Enterococcus* abundance. HGF: Hepatocyte growth factor, IFN: Interferon, IL: Interleukin, LIF: leukemia inhibiting factor, MCP: Monocyte-chemotactic protein, NGF: Nerve growth factor, TNF: Tumor-necrosis factor.

	Group 1	Group 2	Group 3	*p* value* G1 vs. G2/3
IL-1alpha	1.14 (1.14–2.29)	1.14 (1.14–1.14)	1.14 (1.14–1.14)	0.002
IL-4	0.10 (0.10–0.50)	0.10 (0.10–0.20)	0.10 (0.10–0.10)	0.038
IL-12 (p40)	31.52 (9.35–65.23)	19.31 (5.86–43.12)	19.31 (5.86–37.38)	0.075
IL-17	1.69 (0.43–2.89)	0.43 (0.09–1.69)	0.43 (0.09–2.29)	0.038
IL-18	32.04 (19.48–65.45)	20.95 (13.55–27.60)	17.75 (11.07–29.57)	0.007
TNF- alpha	1.86 (0.27–4.11)	0.88 (0.27–2.62)	1.08 (0.27–2.62)	0.057
HGF	2338.31 (947.84–4459.77)	1070.34 (632.39–2174.64)	872.85 (699.38–1746.49)	0.031
IFN-alpha 2	0.01 (0.01–3.10)	0.01 (0.01–0.01)	0.01 (0.01–0.01)	0.015
LIF	11.36 (4.35–26.80)	4.35 (0.46–11.36)	4.35 (0.46–11.36)	0.026
MCP-3	0.43 (0.43–1.46)	0.43 (0.43–0.43)	0.43 (0.43–0.43)	0.033
b-NGF	0.45 (0.16–0.68)	0.12 (0.04–0.45)	0.12 (0.04–0.60)	0.037

Values (pg/ml) shown as median (IQR). *p values (Mann-Whitney U test) adjusted for multiple testing with Benjamini–Hochberg method.

### Microbiota profiles and impaired intestinal barrier translate in a numerical increase of cirrhosis-associated complications

In the overall study cohort, 26.3% (*n* = 25) of the patients died or underwent liver transplantation (LTx) within 90 days (Table 4). Almost a third acquired an infection 30.5% (*n* = 29) with spontaneous bacterial peritonitis (SBP) being the most frequent (*n* = 16; 16.8%) (Table 5, Table 6). Mycotic infections were detected in 6.5% (*n* = 6) subjects within the observational period, with candida esophagitis in 3 cases, urinary tract infection (UTI) in 2 patients, and peritonitis in one case. Moreover, an episode of oHE occurred in 22.1% (*n* = 21) patients and 30.5% (*n* = 29) developed ACLF.

**Table 4.** Incidences of complications during 90 days of follow-up. ACLF: Acute-on-chronic liver failure, LTx: Liver transplantation, oHE: Overt hepatic encephalopathy, SBP: Spontaneous bacterial peritonitis.

	All patients (*n* = 95)	Group 1 (*n* = 39)	Group 2 (*n* = 24)	Group 3 (*n* = 29)
Death/LTx	25 (26.3)	11 (28.2)	7 (29.2)	7 (24.1)
Death	16 (16.8)	5 (12.8)	4 (16.7)	7 (24.1)
LTx	9 (9.5)	6 (15.4)	3 (12.5)	0 (0.0)
Infection	29 (30.5)	14 (35.9)	8 (33.3)	6 (20.7)
SBP	16 (16.8)	8 (20.5)	4 (16.7)	3 (10.3)
ACLF	29 (30.5)	12 (30.8)	7 (29.2)	9 (31.0)
Severe ACLF (≥ grade 2)	12 (12.6)	6 (15.4)	4 (16.7)	2 (6.9)
oHE	21 (22.1)	9 (23.1)	4 (16.7)	7 (24.1)

Values as number (percentage).

**Table 5.** Types of infections during 90 days of follow-up. SBP: Spontaneous bacterial peritonitis, UTI: Urinary tract infection.

	All patients (*n* = 28)	Group 1 (*n* = 14)	Group 2 (*n* = 8)	Group 3 (*n* = 6)
SBP	13 (46.4)	7 (50.0)	3 (37.5)	3 (50.0)
UTI	5 (17.9)	3 (21.4)	0 (0.0)	2 (33.3)
Pneumonia	2 (7.1)	1 (7.1)	1 (12.5)	0 (0.0)
Bloodstream infection	4 (14.3)	3 (21.4)	1 (12.5)	0 (0.0)
Unknown	2 (7.1)	0 (0.0)	1 (12.5)	1 (16.7)
Others	6 (21.4)	3 (21.4)	2 (25.0)	1 (16.7)

Values as number (percentage). Results in more than 100% due to presence of coinfections.

**Table 6.** Infections causing pathogens.

	All patients (*n* = 13)	Group 1 (*n* = 8)	Group 2 (*n* = 4)	Group 3 (*n* = 1)
Enterococcus	5 (38.5)	3 (37.5)	2 (50.0)	0 (0.0)
S. aureus	2 (15.4)	1 (12.5)	1 (25.0)	0 (0.0)
Coagulase negative staphylococcus	3 (23.1)	1 (12.5)	2 (50.0)	0 (0.0)
Candida sp	5 (38.5)	5 (62.5)	0 (0.0)	0 (0.0)
others	2 (1.4)	0 (0.0)	1 (25.0)	1 (100.0)

Values as number (percentage). Results in more than 100% due to presence of coinfections.

**Figure 5.**
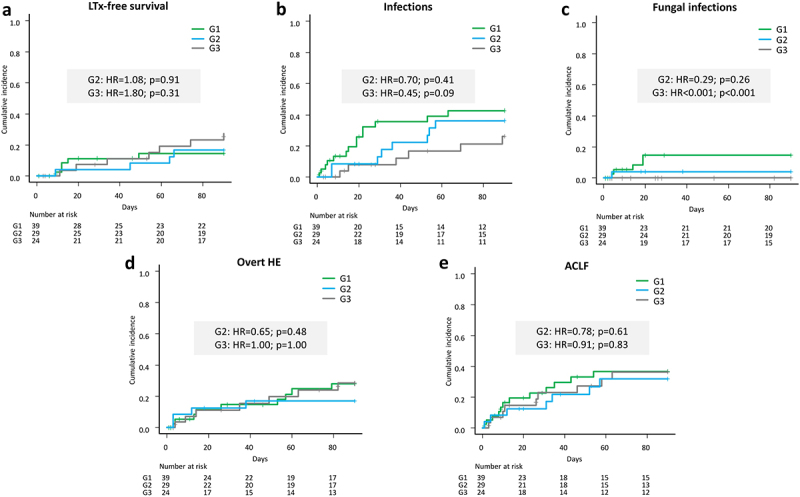
Competing risk analyses of liver transplantation (LTx)-free survival (a), Infections (b), Fungal infections (c), Overt hepatic encephalopathy (d) (oHE) and acute-on-chronic liver failure (ACLF) (e). Group 1 (G1) was treated as reference group.

Regarding LTx-free survival, no differences between groups were detected in the competing risk analyses, treating G1 as the reference group (G2 = HR = 1.08, *p* = 0.91; G3: HR = 1.80; *p* = 0.31) (Figure 5a). Of note, incidences of infections were numerically higher in G1 than in G3 (*n* = 14; 35.9% vs. *n* = 6, 20.7%; HR = 0.45, *p* = 0.09) (Table 4, Figure 5b). Additionally, the risk for fungal infections was significantly increased among G1 patients compared to G3 (*p* < 0.001) (Figure 5c). Contrastingly, the likelihood for oHE (G2: HR = 0.65, *p* = 0.48; G3: HR = 1.00, *p* = 1.00) and ACLF (G2: HR = 0.78, *p* = 0.61; G3: HR = 0.91, *p* = 0.83) was almost equal between groups (Figure 5d,e).

In a second step, the impact of increased Zonulin and elevated sCD163 levels on the disease course were investigated. Although increased Zonulin concentrations were neither linked to inferior survival (HR = 0.81, *p* = 0.75) nor to elevated risk for ACLF (HR = 1.28, *p* = 0.60), numerically higher incidences of infectious complications (HR = 1.83, *p* = 0.16) and oHE (HR = 2.23, *p* = 0.11) were observed in these patients (Supplementary Figure S6). Furthermore, the likelihood for oHE was statistically significantly increased in subjects with elevated sCD163 levels (HR = 3.27, *p* = 0.02) (Supplementary Figure S7).

### Proposal of an integrated model explaining interactions between antibiotic treatment, distinct microbiota profiles and functions, intestinal barrier, inflammation, and infections in patients with cirrhosis

**Figure 6.**
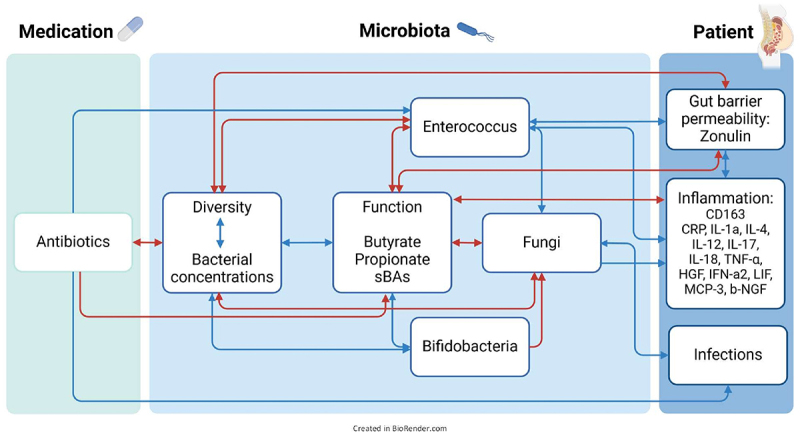
Proposal of an integrated model linking observed results of medication, gut microbiota, markers of intestinal barrier and inflammation and clinical characteristics in cirrhosis patients. Colors of lines represent positive (blue) and negative (red) correlations.

In order to investigate how different parameters of various levels are connected, linear regression analyses were performed and a mechanistic model is proposed in Figure 6. According to our model, antibiotic treatment greatly affected microbiota decreasing bacterial diversity and enriching for *Enterococci*. Concurrently, lower bacterial cell concentrations and increased fungal colonization were observed in those samples. Samples high in *Bifidobacteria* showed opposite patterns, which was also the case for samples of G3. Bacterial key functions, namely the capacity to synthesize SCFA and sBA, were associated with bacterial concentrations and diversity as well as with *Bifidobacteria* (not in the case of sBA), whereas they were negatively associated with *Enterococcus*, fungi, and pro-inflammatory cytokines, the latter showing particularly strong association with butyrate. *Enterococci* and lower capacities to synthesize key metabolites were positively associated with gut permeability (Zonulin) and inflammation markers (sCD163, C-reactive protein (CRP), and various pro-inflammatory cytokines) that were also correlated with each other. Elevated abundances of fungi were linked to CRP and the development of infections; antibiotic treatment was associated with infections as well. *Bifidobacteria* did not correlate with any patient parameters (Figure 6).

## Discussion

Liver cirrhosis is related to high morbidity, poor survival, and accompanied by dysbalanced intestinal microbiota, whose extent proportionally increases with liver disease stages reaching its peak in decompensated cirrhosis.^5,19,20^ Our study confirmed strong dysbiosis in decompensated liver disease patients and additionally provided quantitative insights demonstrating that, next to composition, bacterial load was distinct between patient groups and lower compared to healthy controls (HC). Moreover, we identified medication, specifically antibiotic treatment, as a main contributing parameter explaining the distinct microbial clusters observed, where one particular type comprising a large number of patients was dominated by *Enterococcus* and associated with barrier dysfunction and inflammation. Furthermore, we integrated a major, but so far largely neglected component of gut microbiota, namely, the mycobiome, suggesting an interplay between fungi and specific bacterial taxa that contributes to systemic inflammation and infectious complications.

The gut and the liver are tightly connected via reciprocal organ crosstalk forming the so-called gut–liver axis.^21^ The progression of hepatic disease is, hence, not limited to the liver, but directly influences the intestinal environment and its microbial inhabitants. Typically, a reduction of alpha-diversity which is often associated with increased abundances of pathobionts and lower levels of commensal bacteria has been observed.^22^ Our study supports those results and reiterated a clustering of samples into groups either dominated by *Enterococcus* or *Bifidobacteria* as observed before.^1,5,16^ Those signature taxa correlated with patient parameters, where only the former was linked to barrier malfunctioning and inflammation. Overall, our study supports the general view that *Bifidobacteria* are beneficial, also in the context of chronic liver-disease, and concentrations of this taxon were associated with higher bacterial loads, higher diversity and a lower fungal burden. Regarding the different microbiota clusters that were observed in the studied cohort, antibiotic treatment was revealed as a relevant contributor for *Enterococcus* abundance (discussed in detail below), while lactulose, which was recently shown to promote *Bifidobacteria* growth, did not significantly correlate with abundances of this taxon in our study (*p* = 0.18; data not shown).^5^ This discrepancy might stem from the fact that authors did reveal a correlation only in patients not treated with antibiotics, which was rarely the case for our patients (only 24.7%) as they were more severely diseased and more medicated. However, as a high degree of medication, particularly antibiotic treatment, is common in this highly morbid patient collective, it is essential to analyze the impact of this pharmacological procedure on the intestinal microbiome. It should be noted that patients associated with group 3 (G3) representing the group most closely related to HC displayed the highest diversities and performed best in many parameters including significantly lower levels of Zonulin and sCD163 along with a lower incidence of infections (*p* < 0.1) compared with those of group 1 (G1). Those observations strengthen the common view that microbiota that are most closely resembling those of healthy individuals are the most beneficial, also in the context of liver disease. Hence, manipulations thereof should be done with care (discussed below).

The loss of symbiotic microbes often implicates impaired microbiota functions.^16^ Abundances of pathways related to SCFA synthesis and bile acid transformations were markedly lower in our patient cohort compared with HC, which was particularly pronounced in G1. This observation was recently confirmed on a metabolomic level, where microbiota linked to an *Enterococci* bloom were largely devoid of those metabolites.^16^ SCFA serve as nutrition for colonocytes and are, hence, essential for the maintenance of the intestinal barrier and act anti-inflammatory.^6,10^ Thus, reduced SCFA synthesis directly contributes to barrier dysfunction and increased inflammation as indicated by results of our study. To investigate the impact of microbiota patterns on host barrier functions, plasma Zonulin was measured. Zonulin, a haptoglobin 2 pre-protein, is secreted by intestinal epithelial cells in response to stress inducing factors, mainly due to increased pathobiont abundances.^23^ In line with this, cirrhosis patients exhibited significantly increased Zonulin levels compared to HC in our study. Furthermore, Zonulin correlated positively with *Enterococcus* abundance and negatively with microbiota diversity and SCFA production potential, so that Zonulin levels of G1 exceeded those of the other patients markedly. After secretion, Zonulin upregulates tight junction permeability.^24,25^ Reduced tightness of these intercellular connections allows entrance of gut bacteria-derived products, such as pathogen-associated molecular patterns (PAMPs).^26^ Once PAMPs reach the liver via the portal venous system, liver-resident macrophages (Kupffer cells) quickly respond with the release of sCD163.^27,28^ Importantly, the positive correlation between Zonulin and sCD163 in our study exemplifies the relationship between increased intestinal permeability, translocation of bacterial products and a subsequent pro-inflammatory response. After recognition of microbiota-derived PAMPs by Toll-like receptors, stimulated monocytes and macrophages orchestrate the immune response toward a proinflammatory phenotype via secreting multiple further cytokines beyond sCD163s.^29^ Our observations illustrate a significant correlation between *Enterococcus* abundance, TNF-alpha and IL-1alpha. TNF-alpha reveals an important inducer of the acute-phase reaction in cases of pathogens overcoming structural host defenses.^30^ Increasing TNF-alpha concentrations stimulate IL-1alpha release from macrophages which plays a relevant role in navigating innate as well as adaptive immune responses.^31,32^ By modulating intracellular gene expression, it supports the secretion of IL-17 and several other proinflammatory mediators.^31,32^ IL-17, which finds its main source in Th17-cells and showed highest plasma concentrations in G1-patients, plays a major role in chemokine production and recruitment of neutrophils.^33^ However, activation of Th-17 cells necessitates presence of IL-23.^33^ This cytokine consists of a dimeric structure, including IL-12 (p40) as subunit that also correlated with *Enterococcus* abundance in our study.^34^ Furthermore, by interplaying with IL-18 that also displayed significantly higher levels in G1-patients, IL-12 importantly contributes to the induction of cellular defense.^32,34^ These proinflammatory cytokines furthermore drive activation of CD8^+^ bystander T cells that has been linked with disease severity in patients with decompensated cirrhosis.^35^ Moreover, activation of leukocytes becomes further amplified by PAMP- and cytokine-mediated MCP-3 release.^36^ In their entirety, these factors, beginning with the dysbalanced microbiome and the increased intestinal permeability, create a chain reaction of proinflammatory responses that finally lead to a markedly distinct immune landscape in patients with high *Enterococcus* abundances. Systemic inflammation in turn causes further cellular damage and might therefore accelerate cirrhosis progression and promote organ failure.^37^ Previous studies have linked inflammation to an exacerbation of cognitive impairment and oHE in patients with cirrhosis.^38^ Moreover, systemic inflammation reveals a central element in CAID development that elevates infection susceptibility.^11,12^ In case of infectious complications, increased pathobiont abundances have been discussed as predictor for nosocomial infections in cirrhosis.^39^ Specifically, expansion of *Enterococci* over time has been linked to a higher infection vulnerability among patients undergoing LTx.^16^ In line, G1-patients showed highest incidences of infections during follow-up also in our study.

While previous studies have primarily focused on bacterial components, our study additionally provides detailed insights on the intestinal mycobiome in patients with decompensated cirrhosis. We demonstrated that the extent of bacterial dysbiosis, along with a reduction of cell concentrations, are related to fungal overgrowth in cirrhosis patients. Importantly, the group with the highest intestinal concentrations of fungi indeed had a higher risk for fungal infections. This is of particular clinical importance as mycotic infections, e.g. spontaneous fungal peritonitis, are severe complications, which potentially cause critical illness and end in up to 30–50% fatality.^18,40^ Overall, the bacterial taxon dominating that group, i.e., *Enterococcus* was positively associated with fungi, which was verified by analyzing data from another cohort. However, whether this observation is a result of interkingdom crosstalk or confounded by other factors, such as medication, needs to be elucidated in follow-up studies.

To uncover factors that potentially contributed to the distinct microbial clusters in our cohort, antibiotic treatment was analyzed in more detail. It was found that patients with the lowest cell counts and the highest *Enterococcus* and fungi abundance frequently received broad spectrum antibiotics and higher numbers of different antibiotic types compared with other patients, probably explaining the reduction of autochthonous bacteria observed. Furthermore, some of those drugs, for example meropenem or ceftriaxone, have relatively low or even no efficacy against *Enterococci,* providing selective growth advantages for those bacteria as demonstrated in stratified analyses. A decrease of bacterial concentrations and diversity reduces the competition for nutrients and also diminishes bacterial release of antifungal metabolites, which might result in the observed fungi expansion.^41–43^ Moreover, beta-lactam antibiotics, which were frequently prescribed to G1 patients, have been linked to the release of peptidoglycans from bacteria and a recent study reported enhanced growth of *Candida* in the presence of these substances.^44^ Hence, antibiotic treatment most probably contributed to increased fungi abundances via multiple mechanisms. Our results therefore suggest that antibiotic treatment is a major factor contributing to microbiota damage in patients with advanced cirrhosis. Given the widespread use of prophylactic and broad-spectrum antibiotics in these patients, this observation is of major clinical relevance and suggests to reconsider common practices of ample antibiotic treatments. In summary, multiple treatment of infections with antibiotics might trigger a vicious cycle, where repetitive damage of intestinal microbiota leads to barrier dysfunction and recurrent infections. Hence, indication for antibiotic treatment should be provided carefully in the setting of advanced liver disease. As the missing longitudinal sample collection is a limitation of our study, the regenerative capacity of the gut barrier in end-stage cirrhosis patients needs to be investigated further. Moreover, our results suggest that both cirrhosis and medication contributed to dysbiosis; however, further studies specifically focusing on microbial changes during antibiotic treatment in patients with dLC are required to disentangle in detail cirrhosis-associated microbiota alterations from those induced by antibiotic treatment. Additional limitations are no matching between HC and cirrhosis patients, however, comparisons between cirrhosis patients and HC were not the primary focus of our study. Concerning the numerical differences regarding the clinical complications we cannot exclude that the study cohort might have been too small to reach statistical significance for some endpoints. Major findings on gut microbiota and fungal communities were verified in a validation cohort. However, as our study is the first to connect the different levels verification of findings regarding associations of gut microbiota with inflammation and clinical outcomes were not possible due to the lack of available data in the validation cohort.

Regarding potentially beneficial properties of antibiotics, previous studies have reported that among patients with CHILD C cirrhosis and low ascites protein levels prophylactic treatment with norfloxacin can reduce incidence of SBP and improve one-year survival.^45,46^ The preventive effect against SBP might be due to the reduction of gram-negative *Enterobacteriales* as observed in animal models and cirrhosis patients that received norfloxacin. Contrastingly, the abundances of gram-positive cocci remained stable during treatment.^47,48^ However, we did not find any significant correlation of norfloxacin prophylaxis with microbiota composition in our study and detailed insights into its microbiota modulating effects and potential consequences on intestinal barrier and systemic inflammation in the long term remain to be determined.

In conclusion, distinct individual features of microbiota were present even in the end-stage of liver disease. These were linked to antibiotic treatment, which might represent one important factor for microbiota clustering among patients with advanced cirrhosis. The different microbial patterns were strongly associated with intestinal barrier function and inflammation and linked to cirrhosis-related complications. This opens the door to design novel, microbiota-based treatment options to improve the clinical outcome of patients with decompensated liver cirrhosis.

## Methods

### Study cohort

All patients were recruited from INFEKTA registry, a prospective observational study including consecutive patients with decompensated liver cirrhosis and ascites (German Clinical Trials Registry ID DRKS00010664). Inclusion criteria are evidence of liver cirrhosis, presence of ascites requiring at least one paracentesis and age of 18 or higher. Exclusion criteria were defined as malignant ascites, HIV-infection, congenital immunodeficiencies and presence of any cancer disease, except for hepatocellular carcinoma within the Milan criteria.^49^ Furthermore, patients with a history of organ transplantation, except for those with recurrent cirrhosis after a liver transplant, and complete portal vein thrombosis were excluded. For our analysis, we enrolled all of 95 consecutive patients who were treated at Hannover Medical School between 2017 and 2022 and for whom at least one stool sample was available.

### Clinical data and endpoints

The date of sample collection was defined as baseline. Dietary questionnaires were available for a number of 86 patients. Liver transplantation (LTx)-free survival and occurrence of cirrhosis-related complications were observed during 90 days of follow-up. The following clinical complications were assessed:

- any infections, as described previously^50^
- acute-on-chronic-liver-failure (ACLF), as described previously^51^
- overt hepatic encephalopathy (oHE), according to the West Haven criteria.^52^

### Processing of fecal samples and bioinformatics analyses

DNA was extracted using the ZymoBIOMICS Miniprep Kit (ZYMO, USA) according to the manufacturer’s protocol. For metagenomic analyses libraries were prepared (Illumina DNA Prep, Illumina, San Diego, United States) and subsequently sequenced on an Illumina NovaSeq 6000 in paired-end mode (2 × 150 bp) as described previously.^53^ Quality filtering and removal of human-derived reads from raw reads was done using Kneaddata (Huttenhower lab; v0.7.2; *trimmomatic-options*=“ILLUMINACLIP:/adapters.fa:2:30:10 SLIDINGWINDOW:4:20 minLEN:70)) that were subsequently subjected to metaPhlan4 (reference database: vOct22_CHOCOPhlAnSGB_202212) to obtain taxonomic composition. For determining pathways of specific key functions, namely, production of the SCFAs butyrate and propionate and of secondary bile acids, gene catalogs from UHGG.v2^53,54^representative were used for mapping via BBmap (from JGI; v38.22; paired-end mode).^53,54^ As described previously, average abundances of pathway genes were used to calculate pathway abundances relative to house-keeping genes yielding percentage of bacteria exhibiting respective pathways.^53^ Reconstruction of genomes (metagenome assembled genomes) was done using metaWRAP (v1.3.0) as outlined previously using cut-offs of 80% completeness and 10% contamination.^53^ Genomes were annotated based on the genome taxonomy database (gtdb) using gtdb-tk (v2.1.0; *default mode*).^55^ Detection of *vanA, vanB*, and *vanC* genes in samples was done by ARIBA (v2.14.4 using the CARD database from July 2023). Bacterial load, expressed as bacterial concentration per gram stool, was determined by fluorescent staining combined with flow cytometry as described previously.^53^

For mycobiome analyses, the ITS1 region was amplified based on previously published primers^56^ using a two-step approach as outlined in (annealing temperature was 52°C in first PCR).^57^ Obtained amplicons were sequenced on Illumina MiSeq (2 × 250 bp) as described previously.^53^ Sequences were processed via the DADA2 pipeline (v1.20) in R (4.2.2) and annotated by blasting merged sequences against the UNTIE database (v29.11.22) where 70% identity and 70% query coverage were set as cutoffs. The number of reads assigned as fungal origin was recorded for abundance estimations. For metagenomic-based analyses quality filtered and decontaminated reads were mapped to fungal reference sequences provided by^58^ using BBmap (from JGI; v38.22; paired-end mode), and their relative abundances are expressed as percentage of mapped reads to total reads. Microbiota analyses were performed in all 95 patients and in 19 random healthy controls (HC) who were devoid of any severe liver diseases from a previous study.^9^

For comparison, raw sequence data from a previous study investigating microbiota in cirrhosis patients were downloaded (*n* = 231 (all “visit 1” samples) and *n* = 22 healthy controls) and analyzed as explained above.^5,58^

Hierarchical clustering was based on Bray Curtis (BC) dissimilarities on the genus level using the functions *vegdist* and *hclust* (method=ward.D2) from the vegan package (v2.5.7). Networks were based on Spearman correlations (*p* < 0.01 and Spearman’s rho > 0.3) and were visualized via Cytoscape (v3.7.2). For associations of cytokines with bacterial taxa and fungi relaxed parameters were applied (*p* < 0.05 and Spearman’s rho > 0.2). Phylogenetic trees were constructed from single copy house-keeping genes using gtdb-tk (v2.1.0; default mode). Metric dimensional scaling analysis was done on BC dissimilarities on the genus level using the *phyloseq* package (v1.42.0).^59^ PERMANOVA analyses were performed with the *adonis* function of the *vegan* package (v2.5.7) on BC dissimilarities (genus level;) applying 999 permutations).

### Measurement of plasma Zonulin, sCD163, and cytokine concentrations

Plasma Zonulin, sCD163 and cytokine levels were measured from 82 patient samples and five random healthy controls without severe liver diseases with available plasma samples as surrogate for intestinal permeability and macrophage activation following translocation of bacterial products. Enzyme‐linked immunosorbent assays (catalog number abx151842, abbexa, Leiden, NL and DC1630, R&D Systems, Minneapolis, MN) were performed according to the respective manufacturer’s instructions. For cytokine measurement, a 48 cytokine panel (Bio-Plex Pro Human Cytokine Screening Panel, 48-Plex #12007283, BIORAD, Germany) was utilized.

### Statistical analysis

Baseline characteristics of patients were analyzed using IBM SPSS Statistics (Version 28, IBM®, New York) and R Statistical Software (version 4.2.0, R foundation for statistical Computing, Vienna, Austria) with the “tableone” package. Categorial values are depicted as number and percentage. Chi-Square test was used to compare categorial values between the studied groups. Continuous parameters, shown as median and interquartile range (IQR) were analyzed with Mann–Whitney U test or with Kruskal–Wallis test for comparisons of more than two groups. Cytokine levels of G1 were compared to those of the other patients using Mann-Whitney U test with adjusting for multiple testing using the Benjamini–Hochberg method. For the final model shown in Figure 6 selected patient parameters were correlated with bacterial and fungal features in R using regression analyses (function *lm*) feeding log-transformed data (log(data +1) for continuous variables. Differential abundance calculations of taxa due to antibiotic treatment, as well as comparing untreated samples with HC, linear regression analyses (function *lm*; log(data +1)) were performed and corrected for multiple testing using a local false discovery rate (lfdr) <0.05, calculated via the *fdrtool* package (v1.2.18). Procrustes analysis was done by comparing principal component results (function *rda* from the *vegan* package) based on microbiota data (Hellinger transformed data (function *decostand*) on the genus level) and scaled cytokine patterns (function *scale* from the *dplyr* package (v2.5.0)). The *protest* function (scores = “sites”, permutations = 999) from the *vegan* package was used to test significance. Boxplots were created with GraphPad Prism (version 10.0). Competing risk analyses were done with R commander and plugin “EZR”.

Competing risk analyses were performed to compare LTx-free survival and the cirrhosis-associated complications (treating LTx or LTx and death as competitors, respectively). Patients were censored with the time point of LTx or end of follow-up. In a first step, the clinical outcome of microbiota groups was compared. Here, G1 functioned as reference group. A number of three patients clustered with HC and were therefore not considered for group comparisons. In a second step, the impact of increased Zonulin (median HC Zonulin level fourfold increased) and elevated CD163 concentrations (median HC CD163 level tenfold increased) on clinical complications was analyzed.

## Supplementary Material

Supplemental Material

## Data Availability

Raw sequencing data is available at the European Nucleotide Archive (PRJEB81819). Upon reasonable request, clinical data are available from the corresponding authors.

## References

[1] Acharya C, Bajaj JS. Altered microbiome in patients with cirrhosis and complications. Clin Gastroenterol Hepatol. 2019;17(2):307–21. doi: 10.1016/j.cgh.2018.08.008. 30099098 PMC6314917

[2] Wang Q, Chen C, Zuo S, Cao K, Li H. Integrative analysis of the gut microbiota and faecal and serum short-chain fatty acids and tryptophan metabolites in patients with cirrhosis and hepatic encephalopathy. J Transl Med. 2023;21(1):395. 37330571 10.1186/s12967-023-04262-9 PMC10276405

[3] Solé C, Guilly S, Da Silva K, Llopis M, Le-Chatelier E, Huelin P, Carol M, Moreira R, Fabrellas N, De Prada G, et al. Alterations in gut microbiome in cirrhosis as assessed by quantitative metagenomics: relationship with acute-on-chronic liver failure and prognosis. Gastroenterology. 2021;160(1):206–218.e13. 32941879 10.1053/j.gastro.2020.08.054

[4] Bajaj JS, Heuman DM, Hylemon PB, Sanyal AJ, White MB, Monteith P, Noble NA, Unser AB, Daita K, Fisher AR, et al. Altered profile of human gut microbiome is associated with cirrhosis and its complications. J Hepatol. 2014;60(5):940–947. 24374295 10.1016/j.jhep.2013.12.019 PMC3995845

[5] Odenwald MA, Lin H, Lehmann C, Dylla NP, Cole CG, Mostad JD, Pappas TE, Ramaswamy R, Moran A, Hutchison AL, et al. Bifidobacteria metabolize lactulose to optimize gut metabolites and prevent systemic infection in patients with liver disease. Nat Microbiol. 2023;8(11):2033–2049. doi: 10.1038/s41564-023-01493-w. 37845315 PMC11059310

[6] Wong JMW, de Souza R, Kendall CWC, Emam A, Jenkins DJA. Colonic health: fermentation and short chain fatty acids. J Clin Gastroenterol. 2006;40(3):235–243. doi: 10.1097/00004836-200603000-00015. 16633129

[7] Vital M, Karch A, Pieper DH. Colonic butyrate-producing communities in humans: an overview using omics data. mSystems. 2017;2(6). doi: 10.1128/mSystems.00130-17. PMC5715108 29238752

[8] Singhal R, Donde H, Ghare S, Stocke K, Zhang J, Vadhanam M, Reddy S, Gobejishvili L, Chilton P, Joshi-Barve S, et al. Decrease in acetyl-CoA pathway utilizing butyrate-producing bacteria is a key pathogenic feature of alcohol-induced functional gut microbial dysbiosis and development of liver disease in mice. Gut Microbes. 2021;13(1):1946367. 34369304 10.1080/19490976.2021.1946367 PMC8354657

[9] Kircher B, Woltemate S, Gutzki F, Schlüter D, Geffers R, Bähre H, Vital M. Predicting butyrate- and propionate-forming bacteria of gut microbiota from sequencing data. Gut Microbes. 2022;14(1):2149019. doi: 10.1080/19490976.2022.2149019. 36416760 PMC9704393

[10] Duscha A, Gisevius B, Hirschberg S, Yissachar N, Stangl GI, Dawin E, Bader V, Haase S, Kaisler J, David C, et al. Propionic acid shapes the multiple sclerosis disease course by an immunomodulatory mechanism. Cell. 2020;180(6):1067–1080.e16. doi: 10.1016/j.cell.2020.02.035. 32160527

[11] Albillos A, Martin-Mateos R, Van der Merwe S, Wiest R, Jalan R, Álvarez-Mon M. Cirrhosis-associated immune dysfunction. Nat Rev Gastroenterol Hepatol. 2022;19(2):112–134. doi: 10.1038/s41575-021-00520-7. 34703031

[12] Albillos A, Lario M, Álvarez-Mon M. Cirrhosis-associated immune dysfunction: distinctive features and clinical relevance. J Hepatol. 2014;61(6):1385–1396. doi: 10.1016/j.jhep.2014.08.010. 25135860

[13] Arvaniti V, D’Amico G, Fede G, Manousou P, Tsochatzis E, Pleguezuelo M, Burroughs AK. Infections in patients with cirrhosis increase mortality four-fold and should be used in determining prognosis. Gastroenterology. 2010;139(4):1246–1256.e5. doi: 10.1053/j.gastro.2010.06.019. 20558165

[14] de Franchis R, Bosch J, Garcia-Tsao G, Reiberger T, Ripoll C, Abraldes JG, Albillos A, Baiges A, Bajaj J, Bañares R, et al. Baveno VII – renewing consensus in portal hypertension. J Hepatol. 2022;76(4):959–974. doi: 10.1016/j.jhep.2021.12.022. 35120736 PMC11090185

[15] Moreau R, Jalan R, Gines P, Pavesi M, Angeli P, Cordoba J, Durand F, Gustot T, Saliba F, Domenicali M, et al. Acute-on-chronic liver failure is a distinct syndrome that develops in patients with acute decompensation of cirrhosis. Gastroenterology. 2013;144(7):1426–1437.e9. doi: 10.1053/j.gastro.2013.02.042. 23474284

[16] Lehmann CJ, Dylla NP, Odenwald M, Nayak R, Khalid M, Boissiere J, Cantoral J, Adler E, Stutz MR, Dela Cruz M, et al. Fecal metabolite profiling identifies liver transplant recipients at risk for postoperative infection. Cell Host & Microbe. 2024;32(1):117–130.e4. doi: 10.1016/j.chom.2023.11.016. 38103544

[17] Bajaj JS, Liu EJ, Kheradman R, Fagan A, Heuman DM, White M, Gavis EA, Hylemon P, Sikaroodi M, Gillevet PM. Fungal dysbiosis in cirrhosis. Gut. 2018;67(6):1146–1154. 28578302 10.1136/gutjnl-2016-313170

[18] Hwang SY, Yu SJ, Lee J, Kim JS, Yoon JW, Kim YJ, Yoon J-H, Kim E-C, Lee H-S. Spontaneous fungal peritonitis: a severe complication in patients with advanced liver cirrhosis. Eur J Clin Microbiol Infect Dis. 2014;33(2):259–264. doi: 10.1007/s10096-013-1953-2. 23996048

[19] Ginès P, Krag A, Abraldes JG, Solà E, Fabrellas N, Kamath PS. Liver cirrhosis. Lancet. 2021;398(10308):1359–1376. 34543610 10.1016/S0140-6736(21)01374-X

[20] Qin N, Yang F, Li A, Prifti E, Chen Y, Shao L, Guo J, Le Chatelier E, Yao J, Wu L, et al. Alterations of the human gut microbiome in liver cirrhosis. Nature. 2014;513(7516):59–64. 25079328 10.1038/nature13568

[21] Hsu CL, Schnabl B. The gut–liver axis and gut microbiota in health and liver disease. Nat Rev Microbiol. 2023;21(11):719–733. doi: 10.1038/s41579-023-00904-3. 37316582 PMC10794111

[22] Trebicka J, Macnaughtan J, Schnabl B, Shawcross DL, Bajaj JS. The microbiota in cirrhosis and its role in hepatic decompensation. J Hepatol. 2021;75(Suppl 1):S67–S81. 34039493 10.1016/j.jhep.2020.11.013 PMC8973011

[23] Fasano A. Zonulin and its regulation of intestinal barrier function: the biological door to inflammation, autoimmunity, and cancer. Physiol Rev. 2011;91(1):151–175. doi: 10.1152/physrev.00003.2008. 21248165

[24] Fasano A. Intestinal zonulin: open sesame! Gut. 2001;49(2):159–162. doi: 10.1136/gut.49.2.159. 11454785 PMC1728387

[25] Seethaler B, Basrai M, Neyrinck AM, Nazare J-A, Walter J, Delzenne NM, Bischoff SC. Biomarkers for assessment of intestinal permeability in clinical practice. Am J Physiol Gastrointest Liver Physiol. 2021;321(1):G11–G17. doi: 10.1152/ajpgi.00113.2021. 34009040

[26] Wiest R, Lawson M, Geuking M. Pathological bacterial translocation in liver cirrhosis. J Hepatol. 2014;60(1):197–209. doi: 10.1016/j.jhep.2013.07.044. 23993913

[27] Grønbæk H, Rødgaard-Hansen S, Aagaard NK, Arroyo V, Moestrup SK, Garcia E, Solà E, Domenicali M, Piano S, Vilstrup H, et al. Macrophage activation markers predict mortality in patients with liver cirrhosis without or with acute-on-chronic liver failure (ACLF). J Hepatol. 2016;64(4):813–822. doi: 10.1016/j.jhep.2015.11.021. 26639396

[28] Holland-Fischer P, Grønbæk H, Sandahl TD, Moestrup SK, Riggio O, Ridola L, Aagaard NK, Moller HJ, Vilstrup H. Kupffer cells are activated in cirrhotic portal hypertension and not normalised by TIPS. Gut. 2011;60(10):1389–1393. doi: 10.1136/gut.2010.234542. 21572121

[29] Sweet MJ, Ramnath D, Singhal A, Kapetanovic R. Inducible antibacterial responses in macrophages. Nat Rev Immunol. 2024;25(2):92–107. doi: 10.1038/s41577-024-01080-y. 39294278

[30] Idriss HT, Naismith JH. TNF alpha and the TNF receptor superfamily: structure-function relationship(s). Microsc Res Tech. 2000;50(3):184–195. doi: 10.1002/1097-0029(20000801)50:3<184::AID-JEMT2>3.0.CO;2-H. 10891884

[31] Dinarello CA. Overview of the IL-1 family in innate inflammation and acquired immunity. Immunol Rev. 2018;281(1):8–27. 29247995 10.1111/imr.12621 PMC5756628

[32] Cui A, Huang T, Li S, Ma A, Pérez JL, Sander C, Keskin DB, Wu CJ, Fraenkel E, Hacohen N. Dictionary of immune responses to cytokines at single-cell resolution. Nature. 2024;625(7994):377–384. doi: 10.1038/s41586-023-06816-9. 38057668 PMC10781646

[33] Kolls JK, Lindén A. Interleukin-17 family members and inflammation. Immunity. 2004;21(4):467–476. doi: 10.1016/j.immuni.2004.08.018. 15485625

[34] Hunter CA. New IL-12-family members: IL-23 and IL-27, cytokines with divergent functions. Nat Rev Immunol. 2005;5(7):521–531. doi: 10.1038/nri1648. 15999093

[35] Niehaus C, Klein S, Strunz B, Freyer E, Maasoumy B, Wedemeyer H, Björkström NK, Kraft AR, Cornberg M. CXCR6CD69 CD8 T cells in ascites are associated with disease severity in patients with cirrhosis. JHEP Rep. 2024;6(6):101074. 38882602 10.1016/j.jhepr.2024.101074 PMC11179582

[36] Proost P, Wuyts A, Van Damme J. Human monocyte chemotactic proteins-2 and -3: structural and functional comparison with MCP-1. J Leukoc Biol. 1996;59(1):67–74. doi: 10.1002/jlb.59.1.67. 8558070

[37] Dinarello CA. Proinflammatory cytokines. Chest. 2000;118(2):503–508. doi: 10.1378/chest.118.2.503. 10936147

[38] Shawcross DL, Davies NA, Williams R, Jalan R. Systemic inflammatory response exacerbates the neuropsychological effects of induced hyperammonemia in cirrhosis. J Hepatol. 2004;40(2):247–254. doi: 10.1016/j.jhep.2003.10.016. 14739095

[39] Bajaj JS, Reddy KR, Tandon P, Garcia‐Tsao G, Kamath PS, O’Leary JG, Wong F, Lai J, Vargas H, Thuluvath PJ, et al. Association of serum metabolites and gut microbiota at hospital admission with nosocomial infection development in patients with cirrhosis. Liver Transpl. 2022;28(12):1831–1840. 36017804 10.1002/lt.26552 PMC11097235

[40] Bajaj JS, Reddy RK, Tandon P, Wong F, Kamath PS, Biggins SW, Garcia-Tsao G, Fallon M, Maliakkal B, Lai J, et al. Prediction of fungal infection development and their impact on survival using the NACSELD cohort. Am J Gastroenterol. 2018;113(4):556–563. doi: 10.1038/ajg.2017.471. 29257141

[41] Paterson MJ, Oh S, Underhill DM. Host–microbe interactions: commensal fungi in the gut. Curr Opin Microbiol. 2017;40:131–137. doi: 10.1016/j.mib.2017.11.012. 29175338 PMC5733715

[42] Noverr MC, Huffnagle GB. Regulation of candida albicans morphogenesis by fatty acid metabolites. Infect Immun. 2004;72(11):6206–6210. doi: 10.1128/IAI.72.11.6206-6210.2004. 15501745 PMC523025

[43] Cottier F, Tan ASM, Yurieva M, Liao W, Lum J, Poidinger M, Zolezzi F, Pavelka N. The transcriptional response of Candida albicans to weak organic acids, carbon source, and MIG1 inactivation unveils a role for HGT16 in mediating the fungistatic effect of acetic acid. G3(Bethesda). 2017;7(11):3597–3604. doi: 10.1534/g3.117.300238. 28877970 PMC5677169

[44] Tan CT, Xu X, Qiao Y, Wang Y. A peptidoglycan storm caused by β-lactam antibiotic’s action on host microbiota drives candida albicans infection. Nat Commun. 2021;12(1):2560. 33963193 10.1038/s41467-021-22845-2 PMC8105390

[45] Moreau R, Elkrief L, Bureau C, Perarnau J-M, Thévenot T, Saliba F, Louvet A, Nahon P, Lannes A, Anty R, et al. Effects of long-term norfloxacin therapy in patients with advanced cirrhosis. Gastroenterology. 2018;155(6):1816–1827.e9. doi: 10.1053/j.gastro.2018.08.026. 30144431

[46] Fernández J, Ruiz Del Arbol L, Gómez C, Durandez R, Serradilla R, Guarner C, Planas R, Arroyo V, Navasa M. Norfloxacin vs ceftriaxone in the prophylaxis of infections in patients with advanced cirrhosis and hemorrhage. Gastroenterology. 2006;131(4):1049–1056. doi: 10.1053/j.gastro.2006.07.010. 17030175

[47] Gómez-Hurtado I, Gimenez P, García I, Zapater P, Francés R, González‐Navajas JM, Manichanh C, Ramos JM, Bellot P, Guarner F, et al. Norfloxacin is more effective than rifaximin in avoiding bacterial translocation in an animal model of cirrhosis. Liver Int. 2018;38(2):295–302. 28834270 10.1111/liv.13551

[48] Ginés P, Rimola A, Planas R, Vargas V, Marco F, Almela M, Forne M, Miranda ML, Llach J, Salmerón JM, et al. Norfloxacin prevents spontaneous bacterial peritonitis recurrence in cirrhosis: results of a double-blind, placebo-controlled trial. Hepatology. 1990;12(4):716–724. doi: 10.1002/hep.1840120416. 2210673

[49] Galle P.R. EASL clinical practice guidelines: management of hepatocellular carcinoma. J Hepatol. 2018;69(1):182–236. 29628281 10.1016/j.jhep.2018.03.019

[50] Schultalbers M, Tergast TL, Simon N, Kabbani A-R, Kimmann M, Zu Siederdissen CH, Gerbel S, Manns MP, Cornberg M, Maasoumy B. Frequency, characteristics and impact of multiple consecutive nosocomial infections in patients with decompensated liver cirrhosis and ascites. United Eur Gastroenterol J. 2020;8(5):567–576. doi: 10.1177/2050640620913732. PMC7268939 32213043

[51] Kabbani A, Tergast TL, Manns MP, Maasoumy B. Treatment strategies for acute-on-chronic liver failure. Med Klin Intensivmed Notfmed. 2021;116(1):3–16. doi: 10.1007/s00063-019-00613-x. 31463674 PMC7095250

[52] Vilstrup H, Amodio P, Bajaj J, Cordoba J, Ferenci P, Mullen KD, Weissenborn K, Wong P. Hepatic encephalopathy in chronic liver disease: 2014 practice guideline by the American association for the study of liver diseases and the European Association for the study of the liver. Hepatology. 2014;60(2):715–735. doi: 10.1002/hep.27210. 25042402

[53] Kircher B, Woltemate S, Gutzki F, Schlüter D, Geffers R, Bähre H, Vital M. Predicting butyrate- and propionate-forming bacteria of gut microbiota from sequencing data. Gut Microbes. 2022;14(1):2149019. 36416760 10.1080/19490976.2022.2149019 PMC9704393

[54] Vital M, Rud T, Rath S, Pieper DH, Schlüter D. Diversity of bacteria exhibiting bile acid-inducible 7α-dehydroxylation genes in the human gut. Comput Struct Biotechnol J. 2019;17:1016–1019. doi: 10.1016/j.csbj.2019.07.012. 31428294 PMC6692061

[55] Chaumeil P, Mussig AJ, Hugenholtz P, Parks DH. Gtdb-tk: a toolkit to classify genomes with the genome taxonomy database. Bioinformatics. 2020;36(6):1925–1927. 10.1093/bioinformatics/btz848 PMC7703759 31730192

[56] Hoggard M, Vesty A, Wong G, Montgomery JM, Fourie C, Douglas RG, Biswas K, Taylor MW. Characterizing the human mycobiota: a comparison of small subunit rRNA, ITS1, ITS2, and large subunit rRNA genomic targets. Front Microbiol. 2018;9:2208. doi: 10.3389/fmicb.2018.02208. 30283425 PMC6157398

[57] Rath S, Heidrich B, Pieper DH, Vital M. Uncovering the trimethylamine-producing bacteria of the human gut microbiota. Microbiome. 2017;5(1):54. 28506279 10.1186/s40168-017-0271-9 PMC5433236

[58] Xie Z, Manichanh C. FunOMIC: pipeline with built-in fungal taxonomic and functional databases for human mycobiome profiling. Comput Struct Biotechnol J. 2022;20:3685–3694. 35891785 10.1016/j.csbj.2022.07.010 PMC9293737

[59] McMurdie PJ, Holmes S. Phyloseq: an R package for reproducible interactive analysis and graphics of microbiome census data. PLOS ONE. 2013;8(4):e61217. 23630581 10.1371/journal.pone.0061217 PMC3632530

[60] Buttler L, Velazquez Ramirez DA, Tiede A, Conradi AM, Woltemate S, Geffers R, Bremer B, Spielmann V, Kahlhöfer J, Kraft A, et al. Distinct patterns of microbiota and its function in end-stage liver cirrhosis correlate with antibiotic treatment, intestinal barrier impairment and systemic inflammation. Preprint. 2024; doi: 10.1101/2024.07.27.24311099. PMC12054929 40255076

