# Supplementary material for: Distinct clusters of bacterial and fungal microbiota in end-stage liver cirrhosis correlate with antibiotic treatment, intestinal barrier impairment, and systemic inflammation
Source: Gut Microbes. 2025 Apr 21;17(1):2487209. doi: 10.1080/19490976.2025.2487209 (PMC12054929; doi:10.1080/19490976.2025.2487209)
Supplement: Supplemental Material [file KGMI_A_2487209_SM2328.zip › KGMI Supplement/Revised_manuscript_unmarked_version Supplementary figures.docx]

**Supplementary figure 1.** *Bifidobacteria* were most abundant in G2 (**a**), whereas *Enterococcus* dominated G1 (**b**). Overall bacterial concentrations were decreased in patients, with lowest values in G1 (**c**). Diversity, assessed by observed species (**d**) and Shannon index (**e**), increased from G1 over G2 and G3 to healthy controls (HC). Fungi are largely devoid in HC, but reached high abundances in G1 (**f**).

**Supplementary figure 2.** Metric multidimensional scaling analysis of all samples based on Bray Curtis dissimilarities on the genus level. Samples are color coded according to groupings shown in Figure 1. The three patient samples that clustered with healthy controls (HC) are highlighted in orange.

**Supplementary figure 3.** Microbial key pathways encoding enzymes for the synthesis of secondary bile acids (**a**) and the short chain fatty acids butyrate (**b**) and propionate (**c, d**).

**Supplementary figure 4.** Analysis of metagenomic data from a previously published study on cirrhosis patients (n=231) and healthy controls (n=22). Raw data was downloaded and subjected to the same bioinformatics analyses as performed in our study. Grouping of samples on the genus level based on hierarchical clustering is shown in panel a, along with bacterial composition and abundances of pathways for the synthesis of SCFA and sBA. Measured metabolite concentrations and correlations (Pearson coefficient / Spearman’s rho) with respective pathways is given as well. In panel b abundances of fungi and their taxonomic composition based on custom metagenomic analyses is displayed.

**Supplementary figure 5.** Genera (panel a) and species (panel b) showing significant differences in abundance upon antibiotic treatment. Stratified analyses comparing patient samples treated with any antibiotics (Abx_any) or broad spectrum antibiotics (Abx_broad) with respective untreated patient samples were performed. Furthermore, samples not treated with any antibiotics were compared with healthy controls (Abx_no_vs_HC). The estimate from linear regression analysis is shown, where red refers to increased abundance with Abx treatment and blue signifies lower abundances; in panel b one value for *Enterococcus faecium* (pink (p<0.1)) is shown for comprehensive reasons.

**Supplementary figure 6.** Competing risk analyses of liver transplantation (LTx)-free survival (**a**), infections (**b**), overt hepatic encephalopathy (**c**) (oHE) and acute-on-chronic liver failure (ACLF) (**d**) in patients with and without increased Zonulin levels.

**Supplementary figure 7.** Competing risk analyses of liver transplantation (LTx)-free survival (**a**), infections (**b**), overt hepatic encephalopathy (**c**) (oHE) and acute-on-chronic liver failure (ACLF) (**d**) in patients with and without increased sCD163 levels.
