# Supplementary material for: Distinct clusters of bacterial and fungal microbiota in end-stage liver cirrhosis correlate with antibiotic treatment, intestinal barrier impairment, and systemic inflammation
Source: Gut Microbes. 2025 Apr 21;17(1):2487209. doi: 10.1080/19490976.2025.2487209 (PMC12054929; doi:10.1080/19490976.2025.2487209)
Supplement: Supplemental Material [file KGMI_A_2487209_SM2328.zip › KGMI Supplement/Supplementary table (1).docx]

**Supplementary table 1**

|  | Group 1 | Group 2 | Group 3 | p value* |
| --- | --- | --- | --- | --- |
| IL-1alpha | 1.14 (1.14-2.29) | 1.14 (1.14-1.14) | 1.14 (1.14-1.14) | 0.001 |
| IL-4 | 0.10 (0.10-0.50) | 0.10 (0.10-0.20) | 0.10 (0.10-0.10) | 0.086 |
| IL-12 (p40) | 31.52 (9.35-65.23) | 19.31 (5.86-43.12) | 19.31 (5.86-37.38) | 0.173 |
| IL-17 | 1.69 (0.43-2.89) | 0.43 (0.09-1.69) | 0.43 (0.09-2.29) | 0.088 |
| IL-18 | 32.04 (19.48-65.45) | 20.95 (13.55-27.60) | 17.75 (11.07-29.57) | 0.005 |
| TNF-alpha | 1.86 (0.27-4.11) | 0.88 (0.27-2.62) | 1.08 (0.27-2.62) | 0.148 |
| HGF | 2338.31 (947.84-4459.77) | 1070.34 (632.39-2174.64) | 872.85 (699.38-1746.49) | 0.047 |
| IFN-alpha2 | 0.01 (0.01-3.10) | 0.01 (0.01-0.01) | 0.01 (0.01-0.01) | 0.015 |
| LIF | 11.36 (4.35-26.80) | 4.35 (0.46-11.36) | 4.35 (0.46-11.36) | 0.027 |
| MCP-3 | 0.43 (0.43-1.46) | 0.43 (0.43-0.43) | 0.43 (0.43-0.43) | 0.033 |
| b-NGF | 0.45 (0.16-0.68) | 0.12 (0.04-0.45) | 0.12 (0.04-0.60) | 0.072 |
| IL-1b | 1.18 (0.48-2.30) | 1.18 (0.19-1.82) | 0.94 (0.04-2.31) | 0.501 |
| IL-1RA | 21.99 (6.45-80.16) | 6.45 (6.45-41.14) | 6.45 (6.45-42.31) | 0.457 |
| IL-2 | 0.42 (0.42-0.42) | 0.42 (0.42-0.42) | 0.42 (0.42-0.42) | 0.497 |
| IL-3 | 0.51 (0.33-0.73) | 0.42 (0.15-0.68) | 0.33 (0.15-1.03) | 0.369 |
| IL-5 | 0.73 (0.73-0.73) | 0.73 (0.73-0.73) | 0.73 (0.73-0.73) | 0.240 |
| IL-6 | 4.20 (2.18-8.50) | 2.45 (1.17-4.98) | 2.42 (1.45-5.49) | 0.088 |
| IL-7 | 0.32 (0.32-0.32) | 0.32 (0.32-0.32) | 0.32 (0.32-0.32) | 0.860 |
| IL-8 | 19.67 (7.37-46.94) | 9.88 (5.47-20.86) | 9.88 (7.37-14.32) | 0.068 |
| IL-9 | 102.13 (80.44-128.52) | 106.07 (77.03-134.41) | 106.31 (92.36-116.96) | 0.999 |
| IL-10 | 0.06 (0.06-0.06) | 0.06 (0.06-0.06) | 0.06 (0.06-0.06) | 0.502 |
| IL-12(p70) | 0.52 (0.23-1.06) | 0.23 (0.23-0.66) | 0.52 (0.23-1.06) | 0.544 |
| IL-13 | 0.14 (0.14-0.14) | 0.14 (0.14-0.14) | 0.14 (0.14-0.14) | 0.478 |
| IL-15 | 18.63 (18.63-18.63) | 18.63 (18.63-18.63) | 18.63 (18.63-18.63) | 0.484 |
| IL-16 | 3.41 (3.41-39.73) | 3.41 (3.41-3.41) | 3.41 (3.41-3.41) | 0.094 |
| Eotaxin | 101.00 (75.84-158.30) | 81.33 (45.75-128.66) | 73.52 (41.09-101.49) | 0.039 |
| FGF b | 1.58 (1.58-8.02) | 1.58 (1.58-1.58) | 1.58 (1.58-7.17) | 0.216 |
| G-CSF | 381.50 (195.50-600.52) | 283.26 (177.96-436.67) | 273.54 (164.64-409.10) | 0.098 |
| GM-CSF | 0.14 (0.14-0.57) | 0.14 (0.14-0.57) | 0.14 (0.14-0.14) | 0.732 |
| IFN-g | 33.42 (20.92-47.25) | 34.42 (18.64-48.12) | 27.45 (18.39-45.29) | 0.642 |
| IP-10 | 1210.63 (711.70-2037.68) | 807.32 (640.18-1075.52) | 682.17 (422.68-1212.08) | 0.041 |
| MCP-1 | 1.24 (0.12-3.06) | 0.35 (0.12-2.06) | 0.58 (0.12-1.52) | 0.161 |
| MIP-1alpha | 2.13 (1.43-3.45) | 1.84 (1.22-2.64) | 1.52 (0.72-2.35) | 0.043 |
| PDGF bb | 101.87 (75.41-160.43) | 85.73 (73.33-110.77) | 113.72 (83.68-258.07) | 0.146 |
| MIP-1b | 62.31 (50.82-71.56) | 61.27 (50.24-70.91) | 61.64 (54.99-68.02) | 0.953 |
| RANTES | 311.85 (238.52-456.36) | 261.30 (180.94-434.40) | 285.91 (218.16-343.42) | 0.330 |
| VEGF | 88.74 (51.21-138.86) | 98.41 (49.70-126.97) | 93.64 (28.58-121.46) | 0.797 |
| CTACK | 539.09 (390.25-830.22) | 403.22 (226.44-570.53) | 448.40 (184.45-577.88) | 0.065 |
| GRO-alpha | 144.17 (116.83-177.87) | 125.38 (101.02-178.98) | 132.10 (107.31-157.83) | 0.400 |
| M-CSF | 44.13 (26.05-64.25) | 34.84 (20.56-51.72) | 28.65 (23.30-52.57) | 0.177 |
| MIF | 567.67 (348.26-1140.19) | 415.82 (267.47-640.48) | 369.38 (205.62-536.15) | 0.111 |
| MIG | 573.85 (261.54-1225.85) | 441.68 (134.74-607.53) | 347.22 (139.05-901.64) | 0.253 |
| SCF | 36.67 (25.64-76.03) | 40.91 (21.18-59.53) | 27.34 (12.03-36.67) | 0.105 |
| SCGF-b | 5257.71 (3365.03-8868.85) | 4467.10 (3027.78-6146.59) | 3766.23 (2564.50-5238.92) | 0.143 |
| SDF-1alpha | 635.45 (495.90-1085.12) | 719.11 (407.77-874.79) | 578.81 (332.26-699.50) | 0.265 |
| TNF-b | 35.49 (26.40-45.72) | 35.31 (22.77-46.32) | 38.96 (30.58-41.52) | 0.777 |
| TRAIL | 17.06 (11.76-24.52) | 15.85 (12.46-19.56) | 16.37 (13.24-20.84) | 0.779 |
| IL-2Ralpha | 269.99 (143.37-408.12) | 240.27 (133.43-280.73) | 235.32 (148.89-308.00) | 0.392 |

Values (pg/ml) shown as median [IQR]. * Kruskal-Wallis test.
