# Supplementary figures and images for: Distinct clusters of bacterial and fungal microbiota in end-stage liver cirrhosis correlate with antibiotic treatment, intestinal barrier impairment, and systemic inflammation
Source: Gut Microbes. 2025 Apr 21;17(1):2487209. doi: 10.1080/19490976.2025.2487209 (PMC12054929; doi:10.1080/19490976.2025.2487209)

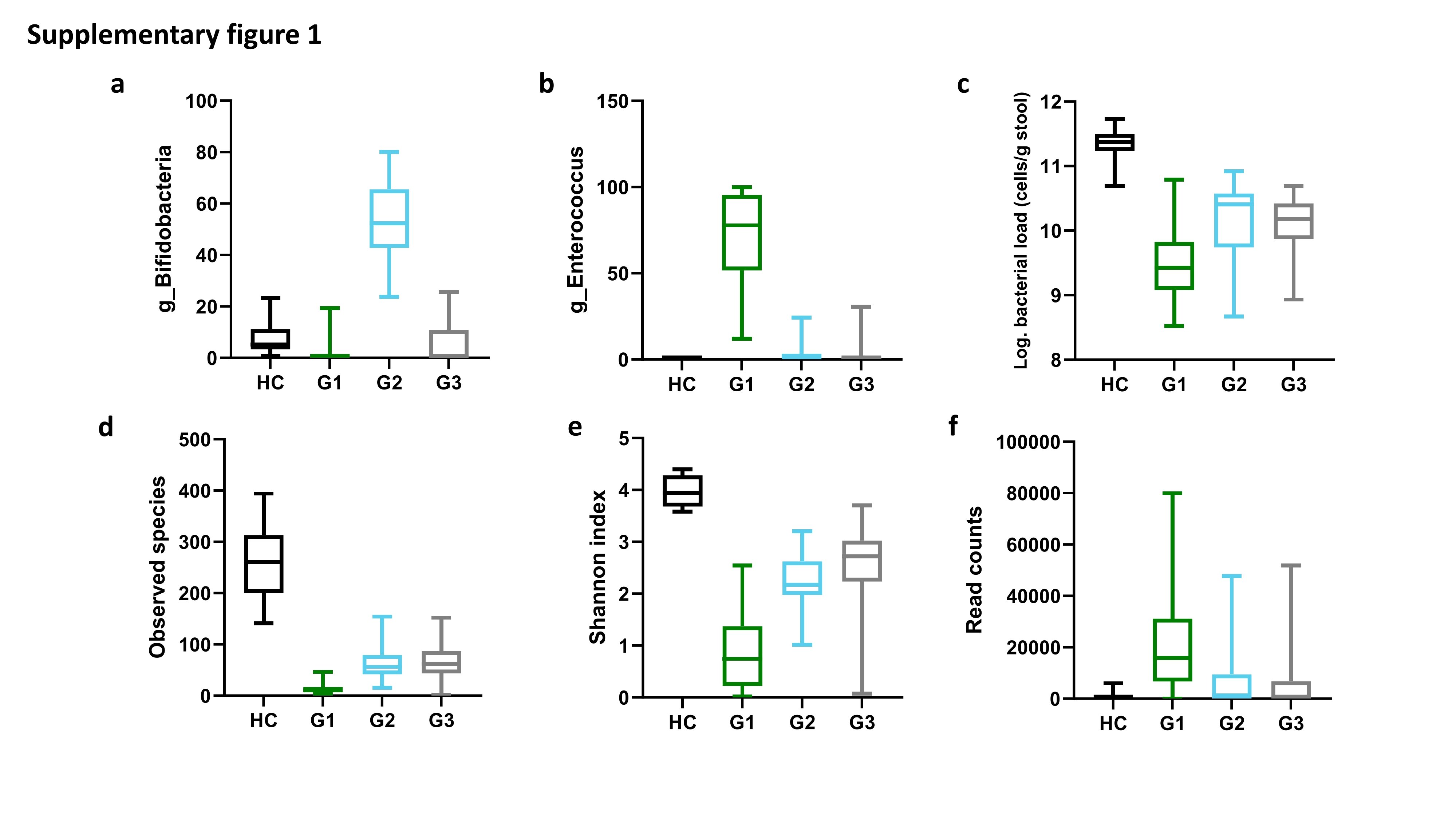

Supplement: Supplemental Material [file KGMI_A_2487209_SM2328.zip › KGMI Supplement/Revised supplementary figures 1.jpg]

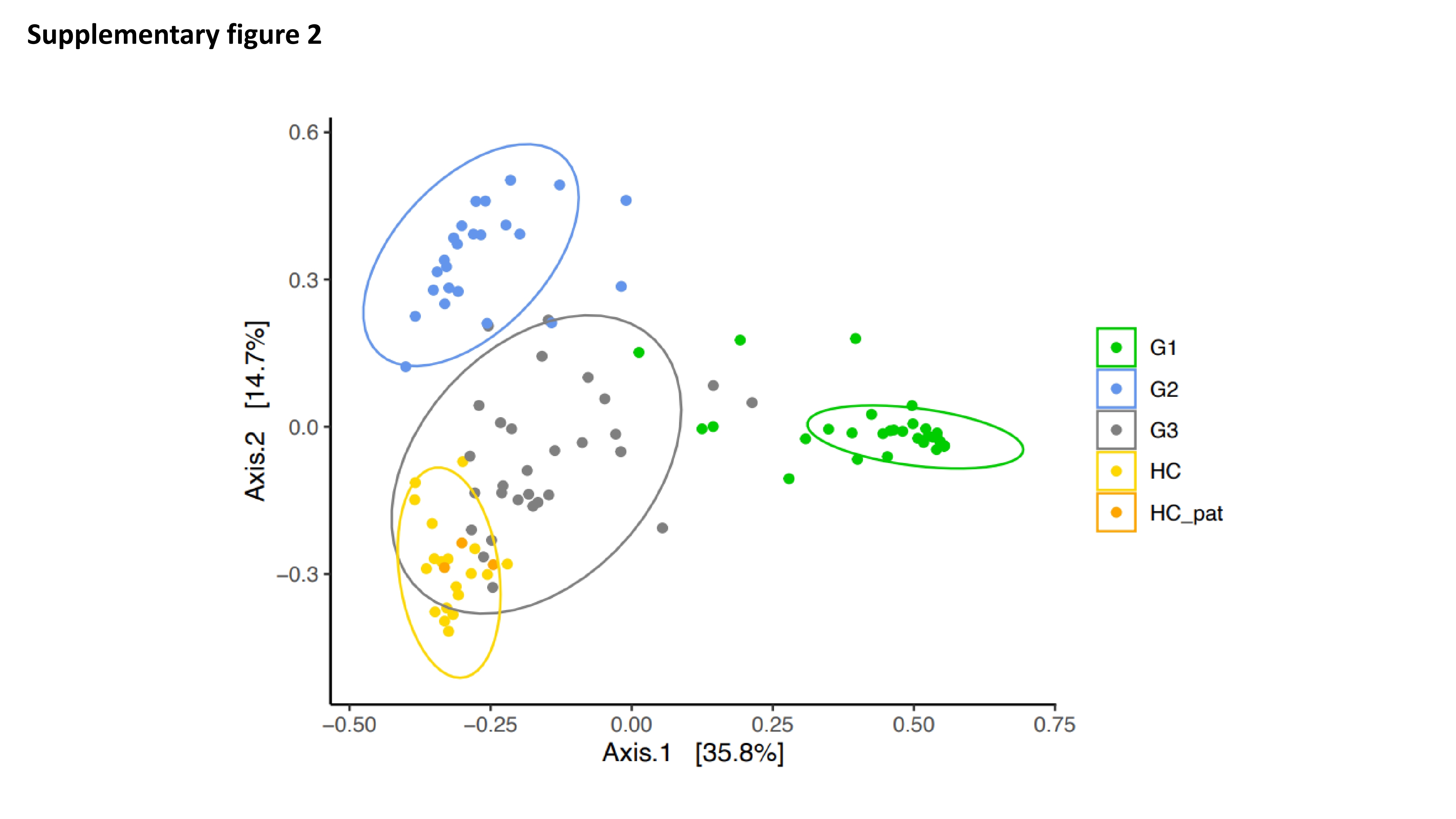

Supplement: Supplemental Material [file KGMI_A_2487209_SM2328.zip › KGMI Supplement/Revised supplementary figures 2.jpg]

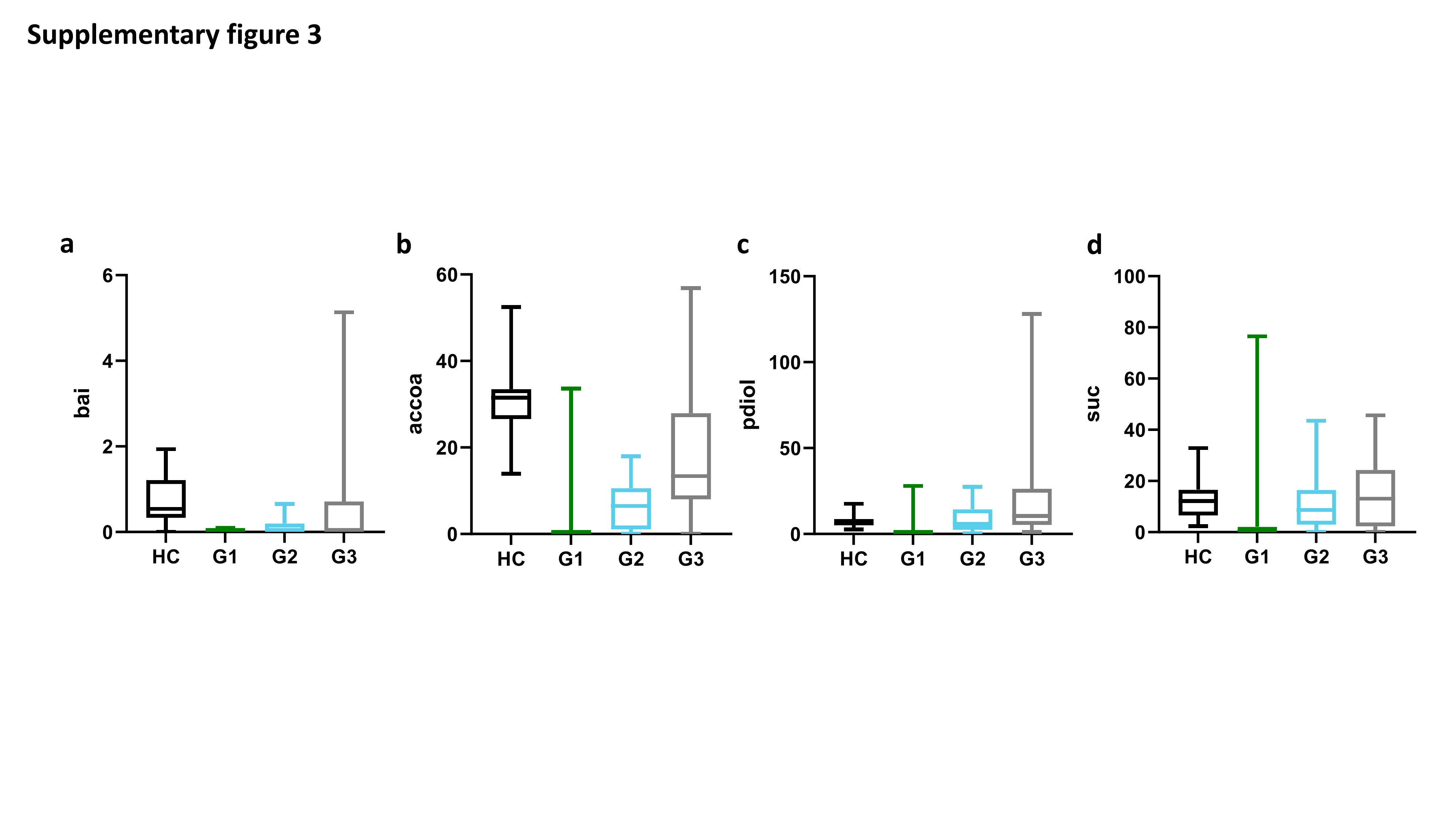

Supplement: Supplemental Material [file KGMI_A_2487209_SM2328.zip › KGMI Supplement/Revised supplementary figures 3.jpg]

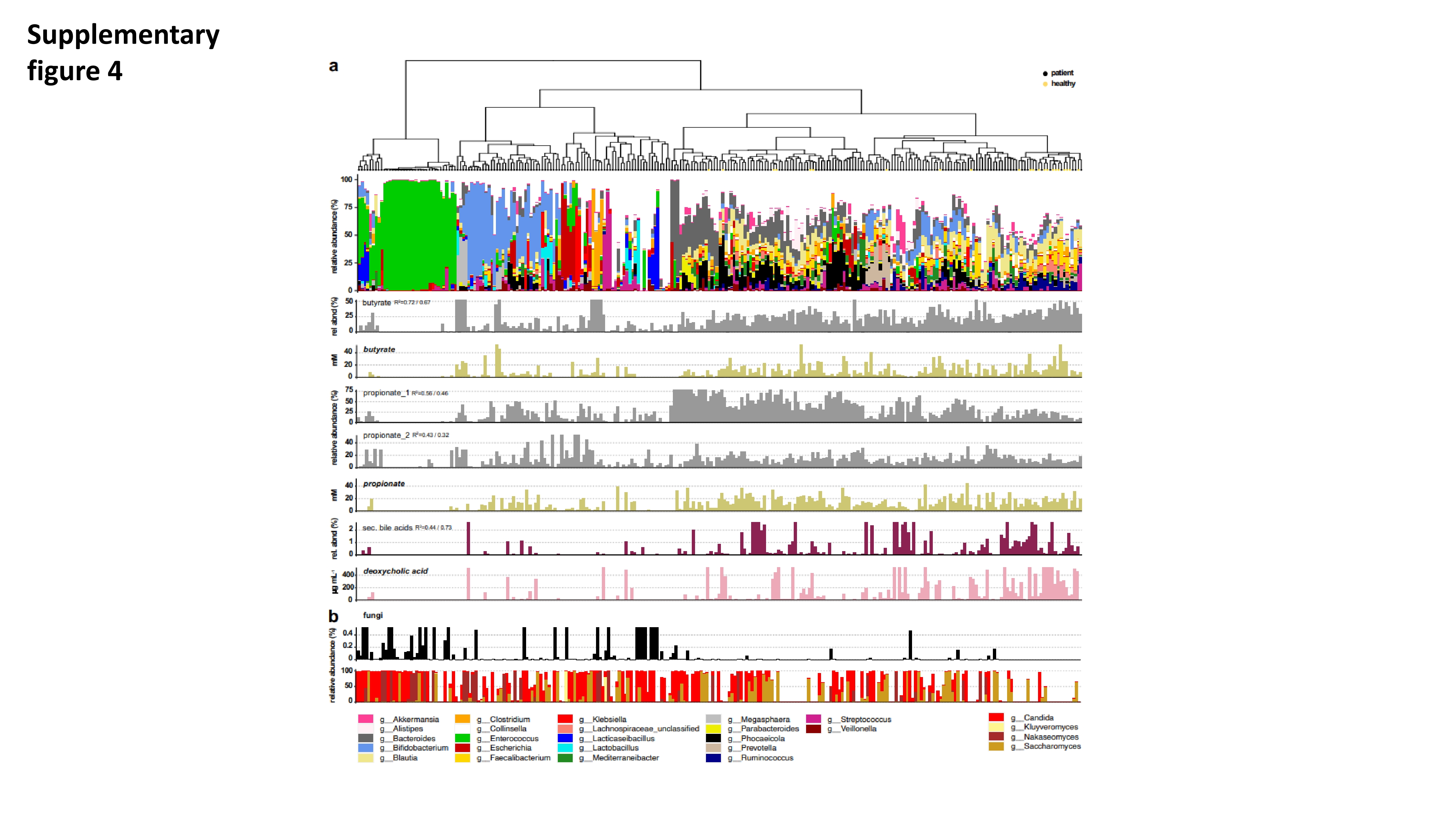

Supplement: Supplemental Material [file KGMI_A_2487209_SM2328.zip › KGMI Supplement/Revised supplementary figures 4.jpg]

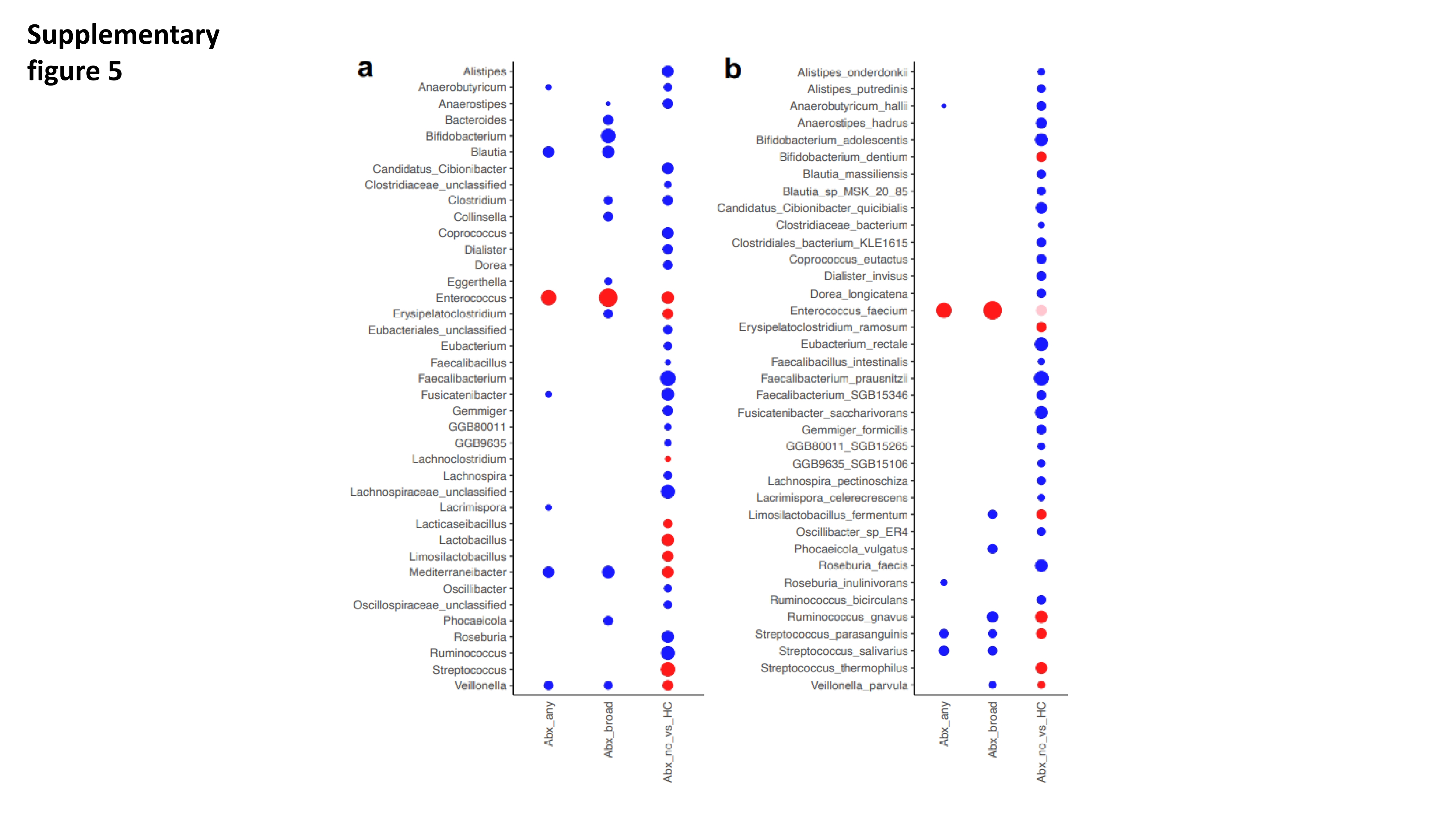

Supplement: Supplemental Material [file KGMI_A_2487209_SM2328.zip › KGMI Supplement/Revised supplementary figures 5.jpg]

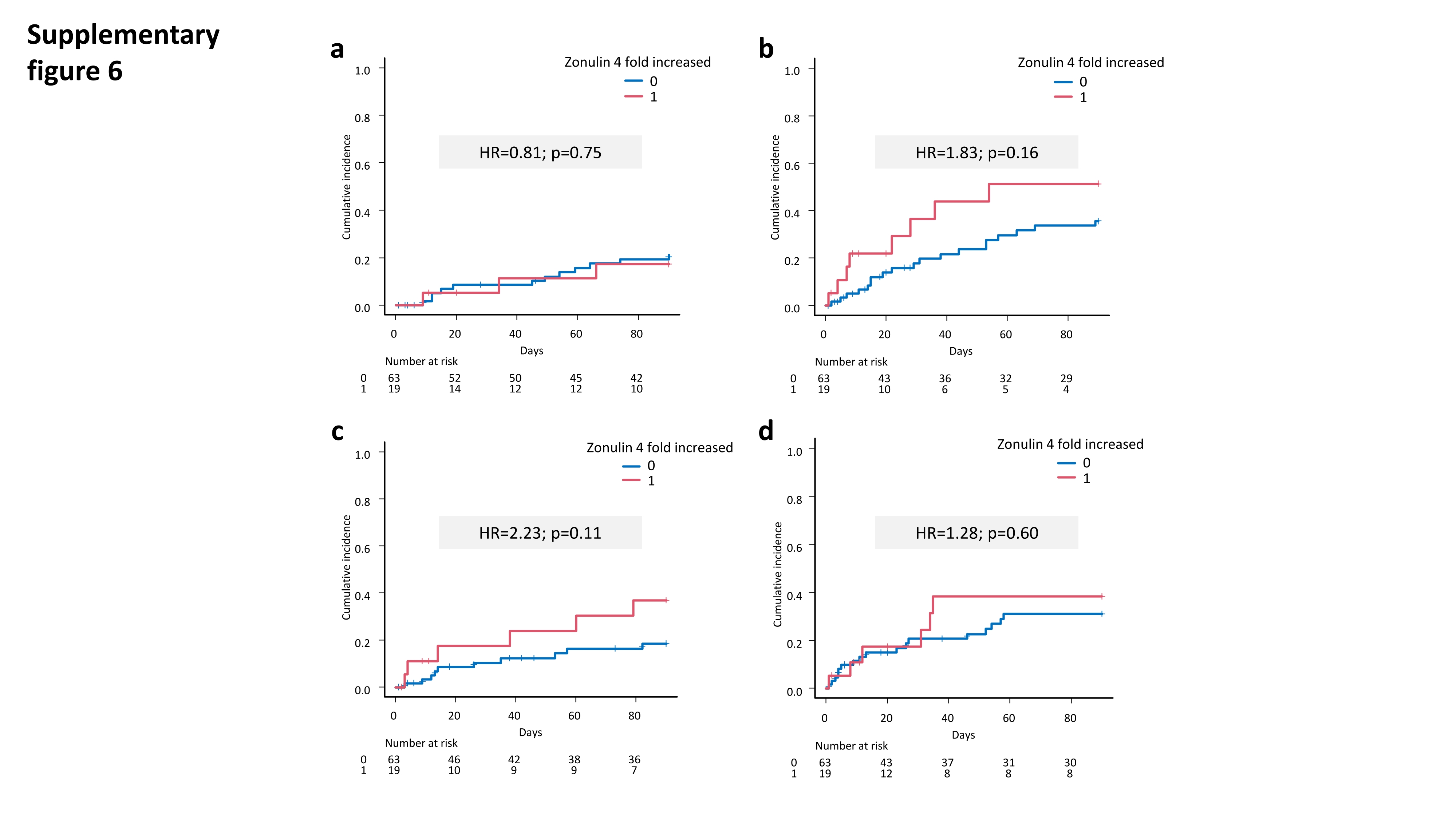

Supplement: Supplemental Material [file KGMI_A_2487209_SM2328.zip › KGMI Supplement/Revised supplementary figures 6.jpg]

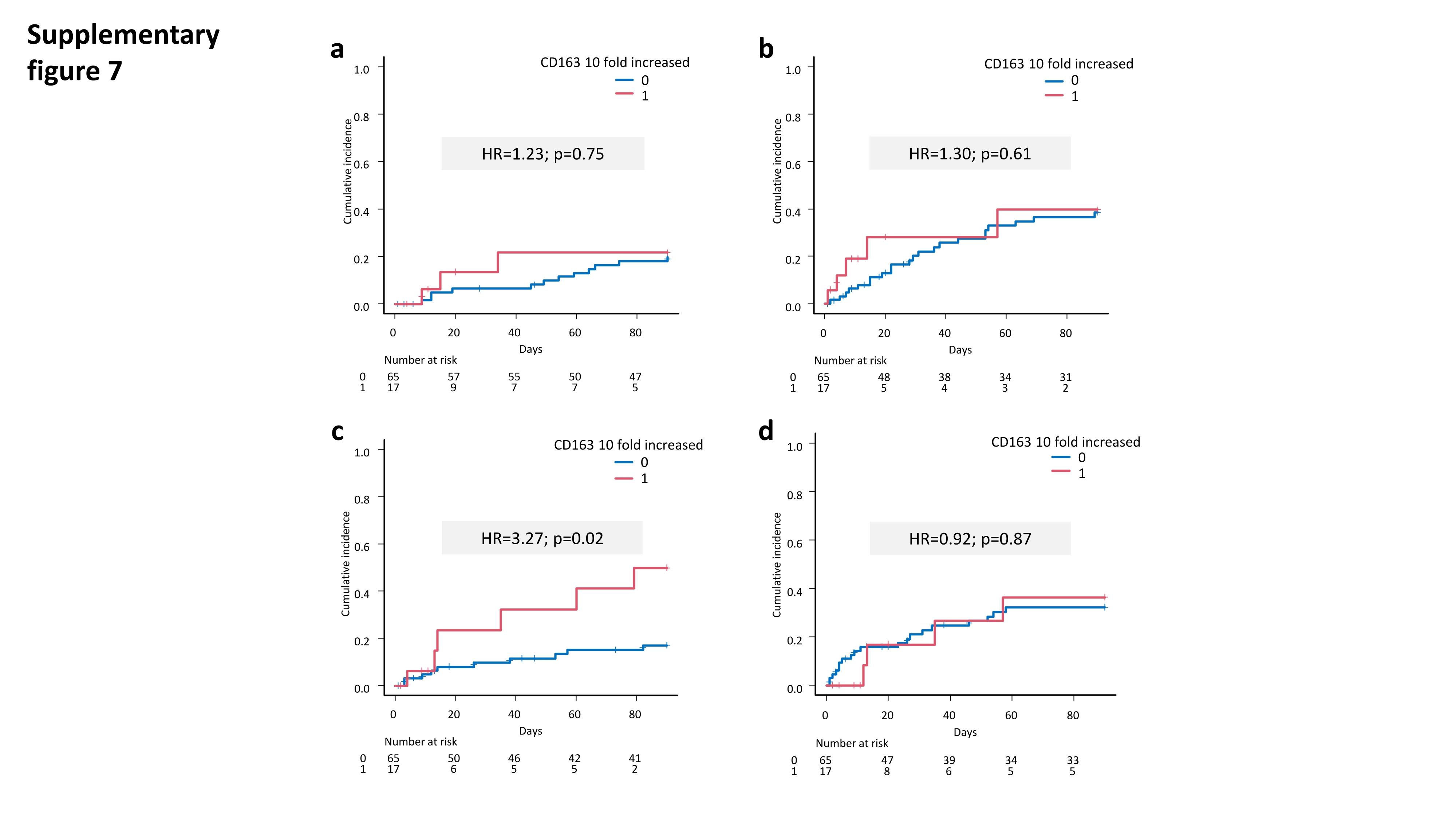

Supplement: Supplemental Material [file KGMI_A_2487209_SM2328.zip › KGMI Supplement/Revised supplementary figures 7.jpg]
